# Identification and characterization of inhibitors of the tuberculosis phosphatase PstP

**DOI:** 10.1016/j.jbc.2026.111316

**Published:** 2026-02-26

**Authors:** Chase Riedel, Jeremy Rahkola, Matthew Reichlen, Hunter Ries, Spencer S. Ericksen, Martin Voskuil, Anthony Gitter, Nathan Wlodarchak

**Affiliations:** 1 Research Service, Rocky Mountain Regional Veterans Affairs Medical Center, Aurora, Colorado, USA; 2 Department of Immunology and Microbiology, University of Colorado Denver, Denver, Colorado, USA; 3 Consortium for Applied Microbial Metrics, Aurora, Colorado, USA; 4 Small Molecule Screening Facility, University of Wisconsin-Madison, Madison, Wisconsin, USA; 5 Morgridge Institute for Research, Madison, Wisconsin, USA; 6 Department of Biostatistics and Medical Informatics, University of Wisconsin-Madison, Madison, Wisconsin, USA

**Keywords:** antibiotics, cell signaling, drug discovery, enzyme inhibitor, high-throughput screening (HTS), kinase, phosphatase, tuberculosis

## Abstract

Tuberculosis is the leading infectious killer worldwide, with two billion infections and 1.25 million annual deaths. Antibiotic resistance drives prolonged, failure-prone treatments, underscoring the need for drugs with new mechanisms. Mycobacterial growth signaling pathways are promising targets. The *Mycobacterium tuberculosis* kinase PknB and phosphatase PstP are essential for growth and virulence. PknB inhibition sensitizes mycobacteria to β-lactams, and we hypothesized that concurrent PstP inhibition would enhance this effect. High-throughput screening identified 126 biochemical PstP inhibitors, four of which showed appreciable growth inhibition. These inhibitors acted additively with PknB inhibitors and displayed enhanced additive effects with β-lactams. PstP inhibition alone was bacteriostatic but antagonized β-lactam activity, consistent with on-target activity. PstP inhibition was additive with kinase inhibition, and inhibition of PstP and PknB with the addition of a β-lactam showed enhanced activity, with some treatment combinations even becoming bactericidal. PstP inhibitors showed low cytotoxicity, low apoptotic activity, and minimal host–cell phosphoprotein effects. Combinatorial treatments have IC_90_ values below individual toxicities, suggesting that effective concentrations of these hits with other inhibitors could be effective at therapeutically relevant concentrations, suggesting high potential for further development and optimization. These represent the first PstP inhibitors with microbiologic activity and demonstrate additive inhibition between β-lactams and kinase/phosphatase targeting. Further development of compounds targeting this pathway may produce an effective tripartite therapy.

Tuberculosis (TB) is the leading cause of death by an infectious disease worldwide, briefly exchanging places with SARS-CoV-2 but then reclaiming its deadly distinction (1). A quarter of the world population is latently infected, and 10.8 million people had an active infection in 2023, resulting in 1.25 million deaths (1). TB is also the leading cause of death for people with HIV/AIDS, with 161,000 deaths in 2023 (1). The COVID-19 pandemic reversed progress made to halt the TB pandemic, with 2021 having the first worldwide increase in TB infections and deaths since 2005 (1). Cases in the United States have also reversed decades of decline, increasing 16% between 2022 and 2023 alone, highlighting the fact that the TB pandemic is worsening everywhere, regardless of a country’s development (2). Antimicrobial-resistant TB is also a significant public health emergency. Approximately 56% of patients with drug-resistant TB do not receive care, and even when treated, these infections carry a 32% mortality rate (1). The duration of treatment is between 6 and 20 months, and limited effective pharmaceutical therapies are available (3). New treatment options are desperately needed to improve patient outcomes and to help end the TB pandemic.

Signaling cascades that utilize phosphorylation are widely successful drug targets for chemotherapies directed against cancer and autoimmune disease (4, 5). Serine/threonine/tyrosine protein kinases (hereafter referred to as kinases) generally have proliferative functions in eukaryotic cells and are classic cancer targets, with over 51 FDA-approved kinase-targeting therapies (6). *Mycobacterium tuberculosis* (Mtb), the primary causative agent of TB, contains 11 kinases, three of which are essential (7, 8). One of these essential kinases, PknB, is a penicillin-binding and serine-threonine-associated (PASTA) kinase (9, 10). PASTA kinases generally serve proliferative roles and respond to cell wall remodeling and growth, changes in amino acid availability, and changes in the cellular environment (11, 12). Several groups developed small molecule inhibitors of PknB; however, reductions in toxicity and improvements in microbiological efficacy are needed (13, 14, 15, 16, 17). Focused medicinal chemistry successfully derived novel PknB inhibitors with exponentially reduced toxicity, and additional optimization is an active area of research in the field (15, 16, 18). These improvements, combined with additional mechanistic strategies, are poised to unlock the potential of phospho-signaling inhibition.

Active TB is not treated with monotherapy due to resistance acquisition, and resistant strains severely limit the combinatorial regimens that can be employed, often with greater toxicity and mixed effectiveness (19). Ideally, newly developed drugs would hit essential targets that are difficult to mutate and synergize together to reduce resistance and increase the therapeutic index. The *Staphylococcus aureus* PknB homolog Stk1 is essential for virulence but is not essential in rich media (20). Genetic deletion of Stk1 results in increased susceptibility to β-lactam antibiotics, such as oxacillin and methicillin (20, 21), and pharmacologic Stk1 inhibition reproduces this phenotype (22). Pharmacologic inhibition of PknB also increases the susceptibility of *Mtb* and other mycobacteria to β-lactam antibiotics (17). This potentiation strategy has additive or even synergistic effects and lowers the dose of kinase inhibitor needed for efficacy, thus widening the therapeutic index.

The serine/threonine/tyrosine phosphatase PstP dephosphorylates PknB and its substrates (23, 24). PstP is known to be essential for growth and virulence, with depletion causing severe cellular defects and pathogen clearance (23, 24). PstP shares 17% sequence identity with the human phosphatase PP2C and shares structural homology with variation in a flap near the active site and by binding a third metal ion (25). The *S*. *aureus* PstP homolog Stp1 is also essential for virulence but is not essential in rich media (20, 26). Genetic deletion of Stp1 in the methicillin-susceptible N315 *S. aureus* strain did not affect β-lactam resistance, but deletion and loss of function of Stp1 in the SF8300ex strain results in an increased resistance to β-lactam antibiotics (27). This is expected since PstP antagonizes PknB activation and substrate activity by dephosphorylation. Interestingly, deletion of both Stk1 and Stp1 in *S. aureus* resulted in an increase in β-lactam susceptibility beyond that of Stk1 deletion alone (28). In *Mtb*, PstP is essential (24); therefore, small molecule inhibitors would be the analogous way to test this phenotype; however, there were no known inhibitors of PstP prior to this work.

Although PstP appears to have some role in increasing β-lactam resistance, lethal doses of an inhibitor should not be able to rescue this phenotype. If it does synergize with a PknB inhibitor and β-lactam drugs, it may be advantageous to use in a tripartite therapy, increasing its therapeutic index. Inhibiting multiple targets is also useful to lower chances of resistance acquisition, and as such, active TB infections are clinically treated with multiple drugs from several classes simultaneously (29, 30) We aim to discover the first small molecule inhibitors of PstP and test the hypothesis that pharmacologic inhibition of PstP will have additive or synergistic effects with a PknB inhibitor and these effects will increase in the presence of β-lactams.

Phosphatases are less studied as drug targets compared to kinases due to their general anti-proliferative functions. Recent small molecules of interest tend to focus on activators (31). As such, there are no libraries of compounds tailored to phosphatase inhibitors. We screened a large (100,629), diverse library using a biochemical assay for PstP activity to find inhibitors. We tested 36 of 126 biochemically validated hits for microbiologic efficacy individually and in combination with a known PknB inhibitor and a β-lactam. We identified four microbiologically active PstP inhibitors, which also have additive effects with a PknB inhibitor, antagonism with a β-lactam, and increased additive effects or synergy with all three together. The phosphatase inhibitors were also less toxic than several known kinase inhibitors and, below their CC_50_, did not induce significant cell-cycle changes in a human lung monocyte cell line. Altogether, this work represents the first known PstP inhibitors with microbiological activity against TB and synergistic effects with kinase inhibition and a β-lactam.

## Results

**Figure 1:**
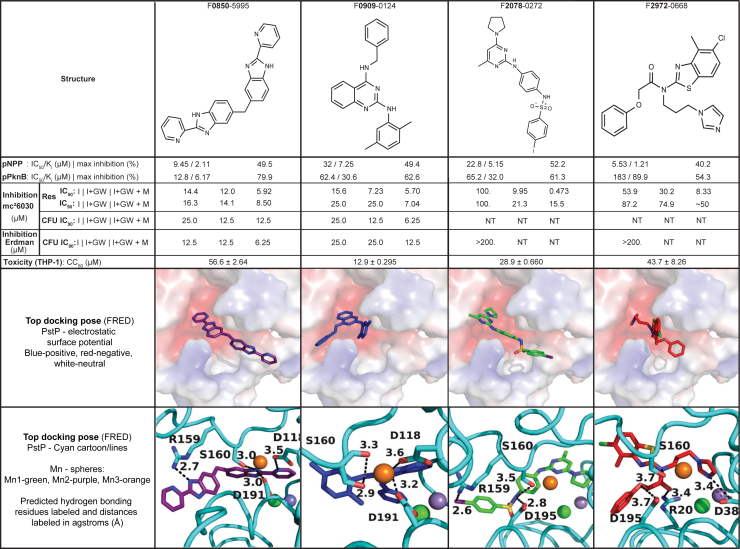
**A summary of the characterization of microbiologically active PstP inhibitors discovered by the biochemical screen.** Compound structures along with IC_50_/K_i_ and % inhibition from the screening assay, IC_50_/K_i_ and % inhibition values from the secondary biochemical assay, IC_50_ and IC_90_ values from microbiological testing, and CC_50_ cytotoxicity values against THP-1 cells were calculated as described in the methods. Consensus docking poses are shown below each compound. Raw data/inhibition curves and SD for biochemical data are presented in Figure S3 and Table S1, and microbiological data are presented in Figures S5 and S6. This figure is reused throughout the manuscript.

Figure 1 is a summary of the key data, some of which is derived from the subsequent figures and some from the supporting information, which contains expanded data or raw data from which these data derive. Data is cross-referenced and indicated in each section and figure.

### A high-throughput screen for PstP inhibition reveals 126 biochemical inhibitors

**Figure 2:**
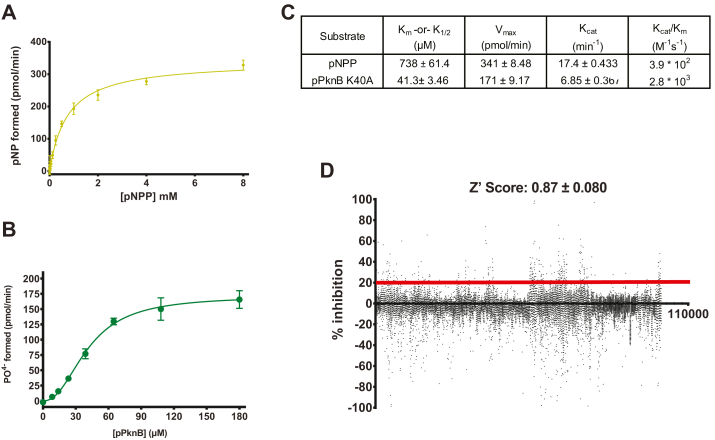
**A biochemical screen of 100,626 compounds discovered 365 compounds with greater than 20% inhibition of the *M. tuberculosis* phosphatase PstP.***A*, the high-throughput assay was developed by determining kinetics for PstP using pNPP as a substrate. PstP 1 to 241 (cytoplasmic domain) dephosphorylated pNPP, and pNP formation was measured colorimetrically by absorbance at 405 nm. The amount of pNP formed was calculated using absorbances of a standard curve of pNP. Experiments were performed in duplicate three times and fit with a Michaelis-Menten model in GraphPad Prism. *B*, a natural substrate assay was developed by determining the kinetics for PstP using phospho-PknB 1 to 331 K40A as a substrate. PstP 1 to 241 dephosphorylated pPknB and free phosphate was separated from the proteins and reacted with a malachite green solution, which was measured colorimetrically by absorbance at 620 nm. Total phosphate released was calculated from absorbances using a standard curve of KH_2_PO4. Experiments were performed in duplicate three times and preferentially fit with an allosteric sigmoidal model in GraphPad Prism. *C*, calculated kinetic parameters for both kinetic assays. *D*, the LifeChem diversity library was screened for inhibitory activity against PstP using the pNPP assay as described in the methods. Cytoplasmic PstP (1-241) was allowed to dephosphorylate pNPP fixed at 2.5 mm with DMSO (positive control), EDTA (negative control) or library compounds and pNP formation was determined as for the kinetic assay in (*A*). Percent inhibition was calculated based on the absorption difference between the positive (100%) and negative (0%) controls. Percent inhibition was plotted across all compounds, and compounds with greater than 20% inhibition (3-sigma, red line) were selected for follow-up.

A biochemical high-throughput screening (HTS) assay was developed to find novel inhibitor hits for PstP. We developed an assay to measure PstP inhibition using purified PstP cytoplasmic domain (1–241) and the universal phosphatase substrate, para-nitrophenyl phosphate (pNPP). pNPP is ideally suited to phosphatase HTS since it is inexpensive, colorimetric, and has a large dynamic range. It was previously shown to be dephosphorylated by PstP (25); however, the kinetics were not fully described nor was it tested for inhibitory assays or any assay in an HTS format. We performed a time course and substrate titration to determine the kinetics of the assay and found that pNPP had a K_m_ of 738 μm and kcat of 17.4 min^−1^ (Fig. 2, *A* and *C*).

The K_m_ is relatively high, but the dynamic range is sufficient at high substrate concentrations. No small-molecule inhibitors were known at the time, making control compounds difficult to ascertain. Aurintricarboxylic acid (ATA) was shown to be a weak inhibitor of the *S. aureus* PstP homolog Stk1 (32); however, ATA was only active against PstP at very high concentrations, making it a weak control (Fig. S1). Sanguinarine is a known inhibitor of PP2C, the closest human phosphatase with identity to PstP at 17% (25); however, it was not active at low concentrations, and at higher concentrations, its strong orange color overlapped with the absorption of the assay product para-nitrophenol (yellow), making it unsuitable as a control. Since a metal cofactor (assumed to be manganese) is necessary for enzymatic activity, the titration of EDTA served as a functional control. The separation of positive (DMSO only) and negative (EDTA) controls yielded a Z′ score of 0.87, making it an excellent assay for HTS. The LifeChem diversity library was chosen for the HTS, containing 100,639 compounds (94,043 unique formulations and 93,948 unique base molecules). The primary HTS was done using pNPP as the substrate and formation of pNP (by absorbance at 405 nm) as the reporter. Compounds were screened as single points with 33 μm of each compound, 2.5 mm of pNPP, and 0.4 μm of PstP 1 to 241 per well. The mean % inhibition was −2.65%, with a standard deviation of 7.52%. Three standard deviations above the mean gave a cutoff of 20% inhibition, which yielded 362 unique active hits (Fig. 2*D*). The full HTS results are available in PubChem with AID# 2060911.

Since inhibitors were initially screened at one concentration, a dose–response curve was needed to confirm hit inhibition and assess which molecules exhibit proper inhibition kinetics. We used the same assay to confirm hits with eight-point curves from 128 μm to 1 μm. Of the 362 hits from the primary screen, 320 (317 unique) were available at the Small Molecule Screening Facility in the quantity and concentration needed for the counter-screen. Hits were confirmed if they had an IC_50_ less than 128 μm (151), a percentage inhibition maximum over 20% (189), and a curve class between 1.0 and 3.0 (209). These criteria resulted in 126 (124 unique) confirmed active hits for a final hit rate of 0.13% (Table S1).

### PstP inhibitors prevent the growth of *M. tuberculosis*

For initial tests of microbiologic activity, we used a high-throughput strategy utilizing the resazurin growth assay and the BSL-2 auxotrophic strain of *Mtb*, mc^2^6030. Thirty-six of the 126 confirmed biochemical inhibitors from the HTS were acquired in quantity based on the highest percent inhibition, lowest IC_50_, curve fit, and availability. Two of the 36 (F6196-0517 & F6200-3415) were chosen as negative controls. Five of the 36 biochemical PstP inhibitors showed microbiologic activity in this assay. One inhibitor, F2971-0726, showed inhibition but did not drop below 10% growth at any concentration tested, and with a minimum inhibitory concentration (IC_90_) of 167 μm (Fig. S2*A*), it was not selected for extensive follow-up. Two compounds, F2078 to 0272 and F2972-0668, had IC_90_s of 100 and 200 μm and two others, F0850 to 5995 and F0909-0124, had IC_90_s of 15 and 29 μm (Fig. S2*A*). These compounds will be subsequently referred to by their first four-digit identifiers. Sanguinarine was used as a control (Fig. S2*B*) as it has known antimicrobial activity (33); and PP2C is the closest eukaryotic homolog to PstP (25). GW779439X, a PknB inhibitor with microbiologic activity (18), meropenem, and isoniazid, were also used as controls (Fig. S2*B*).

### Confirmed hits inhibit the dephosphorylation of pPknB

The HTS and previous work (25) used a synthetic phospho-tyrosine (pY) mimic substrate, pNPP, but *in vitro*, enzymatic activity on a natural PstP substrate is not reported. Using a malachite green solution to detect liberated phosphate is a standard assay in the phosphatase field (34, 35), but peptide substrates are typically used due to the low pH of the detection solution as protein precipitate interferes with the colorimetric measurement. A consensus-binding peptide for PstP is unknown; however, one for a PstP substrate, Wag31 (36), is known for PknB binding (10). PstP was able to dephosphorylate a phospho-threonine (pT) version of this peptide, but unfortunately, the peptide showed substrate inhibition at high concentrations. It is unknown whether this is biologically relevant or an artifact because the full-length substrate was not used. Attempts to make appreciable quantities of full-length phospho-Wag31 *in vitro* were unsuccessful. Phospho-PknB is a known PstP substrate and was ultimately selected for this assay since PknB can be made in quantity and it can reliably phosphorylate itself (37). A catalytically dead (K40A) mutant of the cytoplasmic domain (1–331) of PknB (17) was chosen to minimize potential background enzymatic activity at the high concentrations needed to be used as a substrate or prevent any unforeseen re-phosphorylation of PknB. This was phosphorylated by WT GST-tagged PknB cytoplasmic domain (1–331) and purified to remove active enzyme and ATP. The resulting pPknB was dephosphorylated by the purified WT cytoplasmic domain of PstP (1–241), and free phosphate was measured by the malachite green assay described in the methods (34).

A kinetic analysis of PstP activity (Fig. 2*B*) revealed a significant preference (*p* = 0.0001) for the allosteric sigmoidal model over the Michaelis-Menten model. The hill slope was 2.26 ± 0.33, suggesting catalysis is undertaken in a dimeric complex. Interestingly, pNPP exhibited Michaelis–Menten kinetics (Fig. 2*A*), but PknB is known to dimerize and have allosteric kinetics (17). Therefore, this difference may be due to differences in using a natural substrate or specific to pPknB itself. The k_1/2_ for activity on pPknB is approximately 1.5 logs less than the K_m_ for pNPP, and the catalytic efficiency is nearly a log higher (2.8 × 10^3^) as a result (Fig. 2*C*), suggesting higher specificity for this natural substrate. This efficiency difference between pNPP and a natural substrate is also seen in human PP2C with a two-log improvement (38); however, the natural substrate was a peptide derivative, not full length, which may explain the greater improvement seen in PP2C.

Determining the kinetic parameters allowed inhibition to be more accurately assayed. All four microbiologically active PstP inhibitors had inhibition against pPknB dephosphorylation (Figs. 1, S3). Compound 0850 had the greatest inhibition both in terms of K_i_ (6 μm) and maximum percent inhibition (80%) and showed improved maximum inhibition relative to pNPP dephosphorylation with a comparable K_i_ (Fig. 1). Compounds 0909 and 2078 had reasonable inhibition with improved maximum percent but slightly worsened K_i_ (Fig. 1). The largest contrast was that 2972 was the best inhibitor against pNPP dephosphorylation activity but the worst against pPknB dephosphorylation (Fig. 1). The nonlinear regression for 2972 had a poor fit and high error in determining IC_50_/K_i_ and % inhibition, though it did have some slight inhibitory activity at high concentration as visualized by the plot (Fig. S3).

### PstP inhibitors are predicted to bind at conserved residues in the active site

All ligands in the screening library were docked into PstP (1TXO) using FRED (39), which generally assigned these inhibitors high priority and appeared to place them with a favorable hydrogen-bonding and fit pattern in the active site. Critically, the inhibitors appear to form hydrogen bonds with several residues involved in metal binding in the active site (R20, D38, S160, D191, D118, D195) (Fig. 1). These residues are conserved in the PP2C-like family of phosphatases, and mutations result in loss or reduction in enzymatic function (25). Therefore, it is less likely that mutations in this area would create a viable resistance mechanism. Additionally, this area binds a third metal ion and has a flap region distinct from mammalian PP2C but conserved in related bacteria, which allows for a more specific fit with fewer off-target effects (25).

### PstP inhibitors increase meropenem but not isoniazid resistance

**Figure 3:**
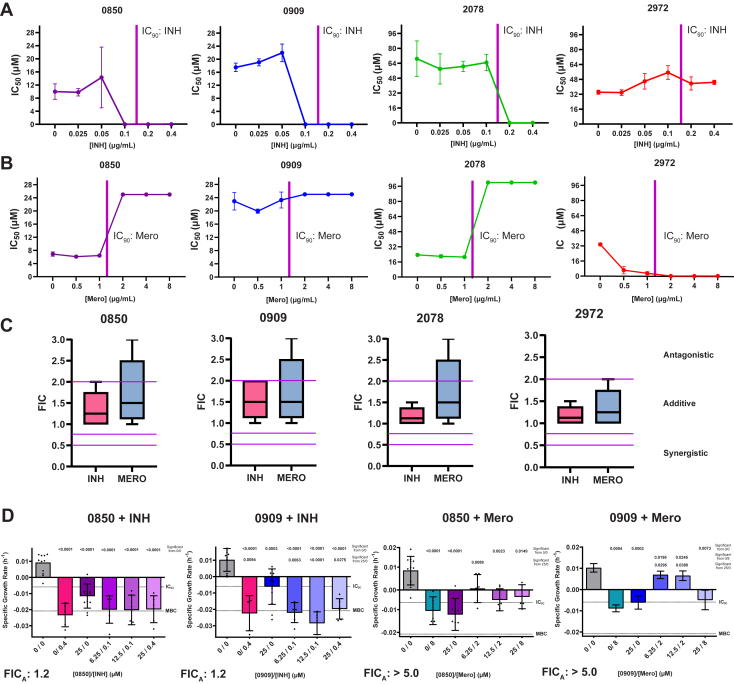
**Meropenem, but not isoniazid, antagonizes PstP inhibitor effects on *M. tuberculosis* (mc^2^6030) activity.** Curves showing the change in IC_50_ of each PstP inhibitor at increasing doses of (*A*) isoniazid or (*B*) meropenem. Compounds were plated in a 2 × 2 matrix serial dilution (checkerboard) series starting at the IC_90_ of each PstP inhibitor and isoniazid or meropenem. Activity curves were determined (Fig. S4) using the resazurin assay described in the methods, and IC_50_ values for each PstP inhibitor were calculated from curves at each dose of isoniazid or meropenem and graphed with error bars indicating standard deviation. If IC_50_ values could not be calculated due to linear (no growth change) or biphasic (increase then decrease) curves, values were called for visualization purposes at 0 (linear & no growth at IC_90_) or at the maximum dose used (biphasic with increased growth at increasing dose). IC_90_ for isoniazid or meropenem alone is indicated with a pink line. *C*, FICs are calculated with Equation 2 (the sum of the ratios of [the effective concentration of PstP inhibitor *with* INH or meropenem over the effective concentration of PstP inhibitor alone] and [the effective concentration of INH or meropenem *with* PstP inhibitor over the effective concentration of INH or meropenem alone]. See methods for details.) and are shown for each isoeffective combination treatment in a box and whisker plot, and ranges corresponding to synergy, additive effects, or antagonism are denoted. *D*, CFU assays were performed with selected combinatorial treatments and specific growth rate calculated (Equation 4) as described in the methods. IC_90_ and MBC are indicated with *dotted lines* to discern bacteriostatic or bactericidal activity. Apparent FIC of each experiment is also indicated below. Individual data points are all technical replicates and independent experiments. One-way ANOVA was used to calculate the significance between different doses in each experiment and *p*-values for significant differences are denoted above each treatment corresponding to the indicated concentration combination at *right*.

Since deletion of Stp1 in *S. aureus* increased vancomycin resistance and loss of Stp1 function increased β-lactam resistance, we hypothesized that partial inhibition of PstP activity would increase resistance to cell wall targeting antibiotics. Since PstP is essential, full inhibition of activity should still result in growth inhibition regardless of other antibiotic presence. To test this, we utilized our screening strain, *M. tuberculosis* mc^2^6030, and the resazurin assay in a checkerboard format, titrating PstP inhibitors against meropenem or isoniazid. We calculated the microbiologic IC_50_ of each compound at each dose of INH or meropenem. We found that the IC_50_ of 0850, 0909, and 2078 did not change with isoniazid nor shift the stand-alone lethal dose (IC_90_) of isoniazid; however low doses of 2972 did antagonize higher doses of INH (Figs. 3*A*, S4*A*). Conversely, we found that in the presence of meropenem, 0850, 0909, and 2078 showed no change in IC_50_ at concentrations of meropenem below its stand-alone lethal dose (IC_90_), but partial PstP inhibition could enhance growth (measured by metabolic activity) even at four times the stand-alone lethal dose (IC_90_) for meropenem (Figs. 3*B*, S4*B*). Fractional inhibitory concentrations (FICs) show additive effects with PstP inhibitors and isoniazid but a broad range of effects, including antagonism, with meropenem (Fig. 3*C*). The IC_50_ of compound 2972 decreased with increasing doses of meropenem and did not exhibit an antagonistic phenotype with meropenem at any dose, possibly suggesting its primary mechanism of action is not through PstP inhibition.

A CFU assay was employed to test whether changes in growth based on metabolism (resazurin assay) translated to changes in bacterial viability. The two most promising inhibitors, 0850 and 0909, were chosen for follow-up since they had the lowest IC_90_s and exhibited the hypothesized meropenem antagonism. Concentrations were chosen based on expected differences from the resazurin growth curves (Figs. S4 and S5) as well as controls at IC_90_. Both compounds inhibited growth individually and were bacteriostatic with significant reductions in growth (Fig. 3*D*). The addition of isoniazid at IC_90_ shifted the effect to more of a bactericidal effect, but only significantly for 0909 (Fig. 3*D*). The FIC for the lowest apparent isoeffective combination (FIC_A_) for each compound was 1.2, indicating additivity within the range measured in the resazurin assay. The same assay was done at IC_90_ with meropenem. Compound 0850 showed a trend towards meropenem antagonism, significant at 6.25 μm, and meropenem antagonism was significant for compound 0909 at 6.25 and 12.5 μm (Fig. 3*D*). Partial inhibition resulted in no difference in growth compared to no treatment despite having meropenem at its IC_90_. No isoeffective concentration tested fell below IC_90_ was at 1 × 0850 or 0909 IC_90_ and 4× meropenem IC_90_, yielding a FIC_A_ of greater than 5.0, suggesting significant antagonism, as seen in the resazurin assay.

### PstP inhibitors have additive effects with a PknB inhibitor and further additivity with a β-lactam

**Figure 4:**
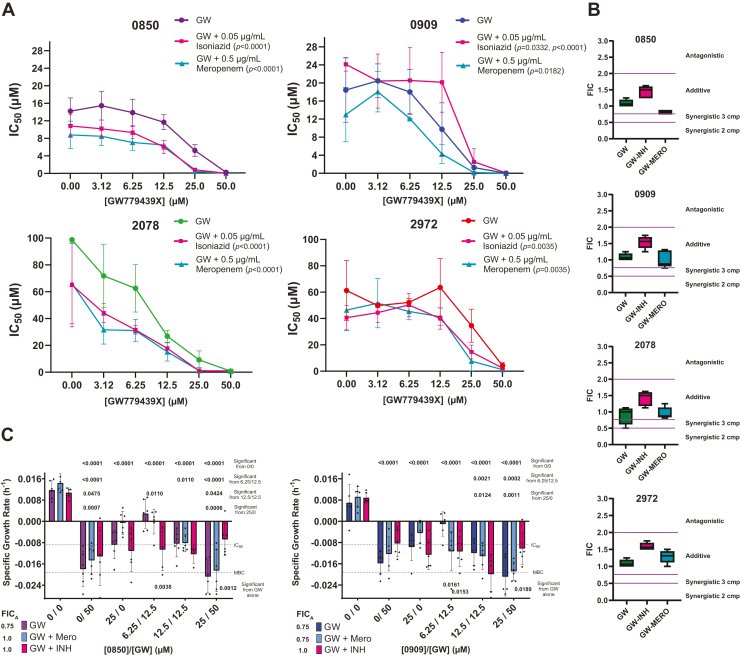
**PstP inhibitors show additive effects on inhibition of *M. tuberculosis* (mc^2^6030) growth when combined with a PknB inhibitor, and this effect is enhanced in the presence of the β-lactam meropenem.***A*, curves showing the IC_50_ of each PstP inhibitor at increasing doses of PknB inhibitor GW779439X in the presence or absence of a sublethal dose of isoniazid or meropenem. Compounds were plated in a 2 × 2 matrix serial dilution series starting at the IC_90_ of each PstP inhibitor and the PknB inhibitor GW779439X (GW) with or without 0.05 μg/ml isoniazid and 0.5 μg/ml meropenem. Activity curves were determined (Fig. S5) using the resazurin assay described in the methods and IC_50_ values for each PstP inhibitor were calculated from curves at each dose of GW and graphed for each concentration with error bars indicating SD. Differences in IC_50_ values for these treatment combinations were analyzed by two-way ANOVA, and *p*-values for significance from GW alone (or after the comma for 0909/INH only from the GW + meropenem treatment) is denoted in the legend. *B*, fractional inhibitory concentrations (FIC) were calculated with Equations 2 or 3 (see methods) for each isoeffective combination treatment and are shown in a box and whisker plot, and ranges showing synergy, additive effects, or antagonism are denoted. *C*, Colony-forming assays (CFU) were performed with selected combinatorial treatments and specific growth rate calculated as described in the methods. IC_90_ and MBC are indicated with *dotted lines* to discern bacteriostatic or bactericidal activity. Individual data points are all technical replicates and independent experiments. Two-way ANOVA was used to calculate significance between treatment groups and *p*-values for significant differences between treatment combinations indicated at *right* are denoted *above* each group. One-way ANOVA was used to compare within-group differences and *p*-values for significant differences between treatment combinations indicated at *right* are denoted *below* each bar. Apparent FIC (FIC_A_, calculated with Equation 2 or 3) for the lowest isoeffective combination from each group is indicated next to the legend.

Since PknB and PstP are both essential in *Mtb* and work together to regulate a significant portion of mycobacterial phosphorylation signaling, we hypothesized that simultaneous inhibition of PknB and PstP would show additive effects. We tested this hypothesis with the same checkerboard assay as with isoniazid or meropenem and found that all four PstP inhibitors showed decreases in IC_50_ (and corresponding IC_90_) at increasing concentrations of the PknB inhibitor GW779439X (hereafter referred to as GW) (Figs. 4*A*, S5*A*). The FICs for isoeffective concentrations had a median of approximately 1.0, suggesting that the effect was additive (Fig. 4*B*: green).

Since deletion of Stk1 in *S. aureus* and pharmacologic inhibition of Stk1 and PknB in mycobacteria increased β-lactam susceptibility, and co-deletion of Stp1 and Stk1 in *S. aureus* increased β-lactam susceptibility beyond that of Stk1 deletion alone, we hypothesized that pharmacologic inhibition of PstP and PknB would show synergistic effects when combined with a β-lactam. To test this, we used the same checkerboard assay but in the presence of a sublethal dose of meropenem (0.5 μg/ml) or isoniazid (0.05 μg/ml), which did not inhibit bacterial growth (Fig. S3, *B* and *C*) or change with PstP inhibition alone (Fig. S4*A*). We found that all four inhibitors showed a significant reduction in growth with meropenem at each concentration of GW tested beyond that of GW alone (Figs. 4*A*, S5*B*). The same pattern was seen for isoniazid, except 0909 had a significant increase in growth at moderate PknB inhibition (12.5 μm) before returning to baseline. This was also seen at the same concentration as isoniazid alone (Fig. S4*A*). FICs were calculated for double and triple combinations as described in the methods. All compounds showed additive effects with GW and meropenem or isoniazid (Fig. 4*B*). Compounds 0850 and 0909 yielded lower FICs with meropenem than with GW or isoniazid, although differences were not statistically significant. Interestingly, additive effects with 2078 or 2972 and GW alone were not enhanced with meropenem or isoniazid.

The CFU assay was also utilized to assess the additive effects of these combination treatments. Concentrations at one-fourth and half IC_90_ for 0850/0909 and GW (6.25/12.5 μm) did not reduce growth to below IC_90_, but the addition of one-fourth IC_90_ of meropenem for 0909 did significantly reduce growth, supporting the resazurin data (Fig. 4*C*). At higher doses, meropenem does not enhance the growth reduction of the 0850/0909 but it is also not detrimental, also supporting the resazurin assay data (Fig. 4*C*). Isoniazid does enhance growth reduction at low doses of 0850/0909 with GW (6.25/12.5), but this effect is not enhanced at increased doses. Conversely, at the IC_90_s of 0850/0909, the combination with GW alone or with GW and one-fourth, the IC_90_ of meropenem is significantly enhanced compared to that with isoniazid or lower concentration combinations, even shifting to a more bactericidal behavior (Fig. 4*C*). Of note, the addition of one-fourth IC_90_ meropenem to only 0850/0909, but not isoniazid, did show a reduction in inhibition, supporting the data in Figure 3, although this was not significant at this lower dose.

### PstP inhibitors are similarly additive with PknB inhibition and antagonistic with meropenem in pathogenic *M. tuberculosis*

**Figure 5:**
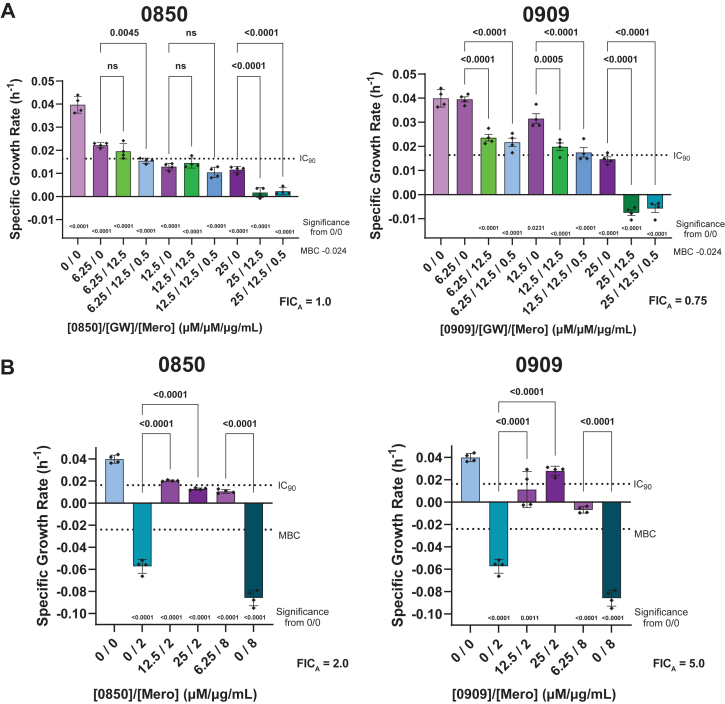
**PstP inhibitors inhibit the growth of pathogenic *M. tuberculosis* (Erdman) and have additive effects with a PknB inhibitor and antagonism with meropenem.***A*, selected concentrations of PstP inhibitors 0850 and 0909 were combined with GW in a CFU assay, and growth was monitored over 192 h (Fig. S6). Specific growth rate was calculated at 96 h, as described in the methods. Doses were compared by one-way ANOVA with *p*-values for significant differences between relevant combinations denoted above and *p*-values for significance from untreated controls noted below. *B*, PstP inhibitors 0850 and 0909 were combined with meropenem in a CFU assay as described above. Individual data points are independent experiments. Significance by one-way ANOVA from meropenem alone treatment is denoted with *p*-values above and *p*-values for significant differences from untreated controls are noted below each treatment.

To assess activity on pathogenic *Mtb*, the four PstP inhibitors active in the resazurin assay, along with GW, were tested in the BSL-3 *M. tuberculosis* Erdman strain using a CFU assay. For the pilot screen, 2078 and 2972 did not show appreciable activity in this strain using the CFU assay; however, 0850 and 0909 showed bacteriostatic growth inhibition up to 8 days of treatment (Fig. S6*A*). Specific growth rate (SGR) was calculated (Equation 4) at the 4-days timepoint to compare treatments. 0909 had an IC_90_ of 25 μm, and 0850 had an IC_90_ of 12.5 μm (Fig. 1). GW had a IC_90_ of 25 μm and appeared bacteriostatic with some bactericidal effects at high concentrations (Fig. S6, *A* and *B*). Meropenem was also tested and showed bactericidal activity at 2 μg/ml (Fig. S6, *A* and *B*). Compounds 0850 and 0909 were also tested in combination with meropenem and with GW in the presence and absence of 0.5 μg/ml meropenem (Fig. S6*A*). Comparing all treatments at the 4-day SGR for 0850 showed that all treatments kept growth below the IC_90_. At low doses, the combination treatment of meropenem and GW significantly decreased growth rate, but at higher 0850 doses, GW was sufficient to significantly reduce growth rate (Fig. 5*A*). The growth rate for treatment with one-fourth and half IC_90_ of 0909 was significantly reduced with one-fourth IC_90_ GW; however, this effect was unchanged with the addition of meropenem (Fig. 5*A*). Further growth reduction was seen at even higher doses (Fig. 5*A*). Both compounds were antagonistic with meropenem with significant reductions in efficacy with all combinations tested (Fig. 5*B*). Compound 0850 abrogated meropenem’s bactericidal activity, but all treatments remained below IC_90_. Compound 0909 behaved similarly, but treatment at IC_90_ for both 0909 and meropenem did increase growth to above the IC_90_, and the SGR was not significantly different from untreated controls. These data support the data seen with the auxotrophic *M. tuberculosis* strain (Figs. 1 and 3), with the differences being GW and 0850 have slightly lower IC_90_s, and meropenem was more bactericidal in the pathogenic strain than the auxotroph.

### PstP inhibitor hits are cytotoxic near or below IC_90_s but impact the cell cycle in human monocytes

**Figure 6:**
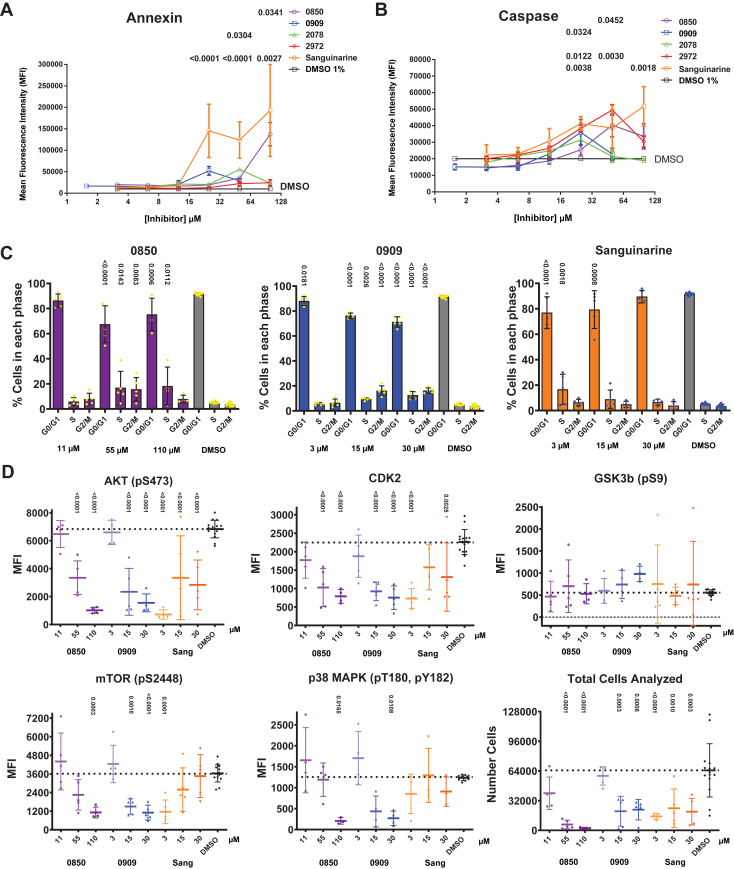
**Microbiologically active PstP inhibitors do not induce appreciable apoptosis or cell cycle changes in THP-1 human monocytes at doses below their CC_50_ and induce variable changes in the cell cycle at higher doses.***A* and *B*, to assess toxicity and apoptosis, THP-1 cells were dosed with PstP inhibitors and stained for viability and annexin V and caspase markers (*panel A*) and ran on flow cytometry (gating shown in Fig. S7). Cytotoxicity was determined from this panel and presented in Figure S8*A* with CC_50_ values listed in Figure 1. Annexin V and caspase signal as a function of dose-response is shown in (*A*) and (*B*), respectively. Significant differences were determined by one-way ANOVA with multiple comparisons by Dunnett’s test and significant *p*-values are indicated above each dose and across from each respective compound. *C* and *D*, To assess changes in specific cell cycle regulators, THP-1 cells were dosed with PstP inhibitors at approximately one-fifth the CC_50_, CC_50_, and 2× CC_50_ for each compound and stained with DAPI to assess the cell cycle stages and five key cell cycle regulation markers: Akt pS437, Cdk2 (total), Gsk3β pS9, p38 MAPK pT180 & pY182, and mTOR pS2448 (*panel B*) and ran on flow cytometry (gating is shown in Fig. S9). Individual data points are all technical replicates and independent experiments. *C*, cell cycle changes for different doses of each PstP inhibitor plus control inhibitor sanguinarine. Significant differences were determined by one-way ANOVA with multiple comparisons by Sidak’s test and *p*-values for significant differences from untreated controls are indicated vertically above each value. *D*, changes in cell cycle markers and total cells were analyzed for each PstP inhibitor plus control inhibitor sanguinarine. Significant differences from untreated controls were determined by one-way ANOVA with multiple comparisons by Dunnet’s test and *p*-values are indicated above.

Early-stage drug development benefits from selecting hits with low toxicity. To determine cytotoxicity, we used the immortalized human monocyte cell line, THP-1. THP-1 cells are frequently used for toxicity studies. Since monocytes differentiate into alveolar macrophages, and *Mtb* resides in these cells during natural infections, they are an ideal choice for this system (40). Early antibiotic development ideas focused on molecules that bound targets with no human homologs. Bacterial kinases and phosphatases do have some homology to human kinases and phosphatases, so evaluating specificity at this early stage will provide valuable information for future development potential. To test beyond simple cytotoxicity, we developed methods to thoroughly assess cellular homeostasis when challenged with these compounds. Since these inhibitors may inhibit human targets, we employed flow cytometry to test for changes in (1) apoptosis markers, (2) cell cycle stages, and (3) phosphorylation of key regulators of the cell cycle with known off-target potential from previous work. We found that the four PstP inhibitors with microbiological function had cytotoxicity ranging from 12.9 to 56.6 μm (Fig. 1) and were similar or less cytotoxic than control human PP2C inhibitor sanguinarine (13.0 μm) and significantly less than CDK2/4 & PknB kinase inhibitor GW (0.00533 μm). Furthermore, PstP inhibitors did not show significant increases in the apoptosis marker annexin V at any concentrations tested except for 0850 at 100 μm and 2078 at 50 μm (Fig. 6*A*), which increased by 13× and 6× respectively. Sanguinarine showed significant increases in annexin V expression at 12 to 19 times untreated controls (Fig. 6*A*). Caspase expression tended to track with dose for all inhibitors; however, this increase was not significant below 25 μm for any compound and most strongly induced by 2972 and control sanguinarine (2.5× each) at higher concentrations (Fig. 6*B*). Taken together, our results suggest these PstP inhibitors generally do not induce appreciable apoptosis signaling at concentrations near their IC90 and are likely not affecting human PP2C or other phosphatases in these pathways.

Low cytotoxicity and few signs of apoptosis were promising results, so next, we wanted to know if we were appreciably changing phospho-signaling, especially with respect to the cell cycle. We chose three concentrations to maximize the chances of seeing changes along the cytotoxicity curve: approximately the CC_50_, two times the CC_50_, and 10% of that concentration. These concentrations land approximately on the toxicity curve’s midpoint (CC_50_) and upper and lower inflection points (Fig. S8*A*). We incubated the compounds with the cells and stained with DAPI to assess cell cycle stages and antibodies to specific cell cycle regulating kinases (pAkt, Cdk2, pmTOR, pGsk3β, and pp38 MAPK α). Flow antibodies to PP2C are not available, but the phospho target on p38 is dephosphorylated by PP2C (41), making it the best available reporter for PP2C activity. We found that 0850 and 0909 have significant effects on the cell cycle by stalling more cells in S or G2/M phase at doses equivalent to or above their CC_50_ (Fig. 6*C*). Sanguinarine seems to have the opposite effect having greater effect on lower doses and less at higher (Fig. 6*C*). Other microbiologically active inhibitors shared the trend between 0850 and 0909 but with somewhat less significance (Fig. S8*C*). High doses of 0850 and 0909 had a significant reduction on phospho-Akt and phospho-mTOR levels as well as a reduction in total Cdk2 expression (Fig. 6*D*). Phospho-Gsk3β and p38 MAPK levels were less affected (Fig. 6*D*). This trend tended to hold for other compounds and throughout all stages of the cell cycle (Fig. S8, *D*–*F*). In general, there appeared to be more variation in the expression of all five markers among cells at lower doses of compound; however, due to some toxicity of the compounds at doses near or above their CC_50_, fewer cells were analyzed at higher concentrations (Figs. 6*D* and S8*B*).

## Discussion

Protein phosphatases have long been viewed as “undruggable” targets. This primarily stems from the fact that most human phosphatases are involved in anti-proliferative mechanisms, making them counterproductive candidates for cancer drugs. Additionally, many eukaryotic phosphatases share a small number of catalytic subunits, having evolved diverse holoenzyme combinations to regulate many disparate processes (42), Because the same catalytic subunit is used along with different regulatory subunits, any small molecule targeting a catalytic site would impact multiple functions and pathways. Bacterial S/T/Y protein phosphatases were discovered relatively recently, and characterizing them is still ongoing. The *Mtb* phosphatase was identified as an essential gene product, which suggested that it may be a good drug target (24). TB is notorious for some fraction of *Mtb* having intrinsic resistance to at least one if not more of the clinically used antibiotics. As such, active disease is never treated with monotherapy (19). Data from *S. aureus* on its homologous phosphatase (Stp1) and kinase (Stk1) suggested that knocking out both had synergistic effects, especially with regard to increased β-lactam susceptibility (28). Kinase/β-lactam synergy was known from our and others’ previous work in *S. aureus* and *Mtb* (17, 20, 21, 22), therefore, we hypothesized that pharmacologically inhibiting the phosphatase would be of additive benefit with PknB inhibition, and this would be an excellent candidate pathway on which to develop drugs that work together.

We knew drug discovery efforts for PstP would require novel inhibitor hits. Since phosphatases are not commonly targeted, no target-specific compound libraries exist. Therefore, a large, diverse library would be needed to ensure a high likelihood of obtaining sufficient hits. This meant prioritizing a high-throughput assay that could be run cost-efficiently with many compounds and a large dynamic range to accurately capture the most hits possible. The results from the screen were promising, albeit with a lower hit rate than expected. A 20% cutoff was chosen for activity, equivalent to three standard deviations above the mean. Many compounds had “negative” activity in the assay, and although some could be “activators,” most were likely colorimetric interference with the assay from strongly colored compounds like sanguinarine. Therefore, this assay may have a higher number of false negatives than typical for HTS, but the alternatives to tease that apart would have a low return on investment. Interestingly, sanguinarine does not inhibit PstP enzymatic activity at concentrations which do not interfere with the assay, but curiously, it does inhibit bacterial growth at high concentrations (Fig. S2*B*). Sanguinarine had previously known antibacterial properties (33), but this was never connected to phosphatase inhibition. Recently, novel derivatives of sanguinarine were shown to have anti-tuberculosis activity, and although the mechanism was not known for this discovery, it is possible that some PstP inhibition could be involved (43).

A qualitative assessment of the 126 confirmed hits revealed that a threefold or pseudo-threefold symmetry axis was a common but not universal theme (Fig. 1 & Table S1). Coincidentally, the Stp1 inhibitor aurintricarboxylic acid (ATA) (Fig. S1) also possesses this symmetry (32). The predicted binding pocket of PstP consists of a deep T-shaped groove (Fig. 1), which may be the reason for this theme. These inhibitors are also predicted to bind near the metals in the active site of PstP (Fig. 1). The binuclear metals (green and purple in Fig. 1) are necessary for catalysis for the PPM family of phosphatases, with M2 (purple) as critical for substrate binding and the rate limiting factor in catalysis for human PP2C (38). Our inhibitors appear to bind closer to M3, and disrupting M3 binding is also associated with catalytic defects (25, 44). The inhibitors are predicted to interact with several of the residues that coordinate active site metals, and although currently unknown, a dual mechanism of substrate blocking and metal disruption could be beneficial to lowering resistance acquisition potential. The mechanism of binding and prediction of resistance mutants, if any, is the focus of future work.

We found that five out of the 36 inhibitors tested for microbiologic activity showed activity, with four reducing growth to nearly 0% (Fig. S2). Three of them had antagonistic effects with meropenem and not isoniazid (Figs. 3 and 5). All four had additive effects with GW, two of which were enhanced with the addition of meropenem (Figs. 4 and 5). Collectively, these data support the hypothesis that inhibition of PstP *in vitro* has additive effects with a PknB inhibitor and is antagonistic with a β-lactam. Compound 2972 does have additive effects and is a reasonable biochemical PstP inhibitor, but it does not have the antagonism with meropenem predicted and seen with the others. This might suggest that 2972 is also acting off target as well, but it is also the least microbiologically active, and it was the only inhibitor to show poor activity against PstP dephosphorylation of the natural substrate pPknB, so it is possible it is not inhibiting PstP at the level needed to see antagonistic effects. These patterns suggest that the antagonistic phenotype likely reflects broader network-level perturbations rather than simple target engagement. This is consistent with PstP’s role in general antibiotic tolerance (45). Although effects were generally additive rather than synergistic, they were consistent and reproducible across different experimental models.

Based on *S. aureus* knockouts and previous kinase-β-lactam synergy data (17, 22, 28), we hypothesized we would observe stronger effects. It is possible that compound class variations for the kinase inhibitor have a role, for example, GSK690693 has very weak activity on its own against mycobacteria *versus* GW779439X (17, 18). This could be due to bacterial entry differences, and it is possible a β-lactam is modulating entry, allowing for a more potent compound (GSK690693) to inhibit more efficiently and thus show greater synergy. Mycobacteria have 4 to 24 S/T kinases (*Mtb* has 11) compared to two in *S. aureus* (8), and it is possible that redundant pathways reduce the synergistic potential in mycobacteria, but if true, improvement may be seen with dual-specificity kinase inhibitors (16). The differential interaction profiles with β-lactams *versus* isoniazid suggest pathway-specific regulation by PstP inhibition; however, this is inferred based on the expected antagonistic/additive phenotype, and evidence that these compounds are directly and primarily working through PstP is difficult to obtain. Since PstP is essential, and engineered *M. tuberculosis* that overexpresses or knocks down expression of PstP both have negative effects on cell growth (24), treating these bacteria with PstP inhibitors would show the same growth defect phenotype and not provide definitive evidence of a direct action through PstP. Identifying resistance mutations in PstP could provide direct evidence of *in vivo* interaction; however, our docking analysis predicts that these inhibitors bind in highly conserved regions of PstP’s active site, and known *in vitro* mutations in this area have a catalytic detriment (25, 44) and catalysis is essential for survival (24), therefore these mutations are unlikely to rise spontaneously, and engineering *M. tuberculosis* with a mutants that would abrogate binding while maintaining wild-type catalysis and growth would be challenging. Although these are difficult limitations in gaining a definitive interpretation of the *in vivo* data, the essentiality and conserved binding regions further highlight the potential of this drug target. Future medicinal chemistry development will provide tools to probe these questions more directly by using a panel of related compounds to assess differences in biochemical and microbiological activity. Furthermore, the mechanism of kinase- β-lactam synergy and by which phosphatase inhibition antagonizes β-lactam activity but is enhanced with kinase inhibition is still unknown. This work now provides critical tools necessary to probe this mechanism and that is a focus of ongoing work.

One of the most promising developments with the PstP inhibitors was discovering their relatively low cytotoxicity (Figs. 1, S8*A*). Ours and others’ previous work with PknB inhibitors regularly suffered from high toxicity with initial hits (18, 46). This isn’t surprising since most kinase inhibitor libraries are comprised of many failed chemotherapy compounds for kinases implicated in cancer and were developed for human cell targets. Although efforts to lower PknB inhibitor cytotoxicity by several logs were successful, the best improvement was still in the low micromolar range (18). The initial hits for PstP inhibitors presented here already started with double-digit micromolar toxicities (Fig. 1), which is significantly above even the best PknB inhibitors. Furthermore, they do not appreciably activate apoptosis pathways at levels near their microbiologic IC_90_ (Fig. 6, *A* and *B*). These PstP inhibitors appear to alter the cell cycle at higher doses, causing a stalling in the S and G2/M phases (Figs. 6*C* & S8*C*). Interestingly, doses of 0850 and 0909 at or above their CC_50_ do show some reduction in phospho-Akt, phospho-mTOR, and total Cdk2 (Fig 5*D* and S8, *D*–*F*). This may be due to global pathway disruptions as cells are dying or some specific inhibition, and follow-up work will investigate this phenomenon. Surprisingly, changes in p38 MAPK activation (pT180 and pY182) were not particularly significant in G0 but were in S and G2/M, and the trend was downward with dose (Fig. S8, *D*–*F*). PP2C directly dephosphorylates these residues (41), suggesting that PstP inhibitors do not inhibit PP2C in monocytes. Known PP2C inhibitor sanguinarine did not significantly change p38 phosphorylation; however, the trend is upward with dose as would be expected if it was inhibiting PP2C (Fig. S8, *D*–*F*). Collectively, the low cytotoxicity, apoptosis activation, and lack of major disruptions to the cell cycle and cell cycle markers at near-bacterial IC_90_ doses make these compounds very attractive starting points for further hit-to-lead optimization, and future work will focus on this process.

Overall, this work represents a major step forward in our understanding of the importance of protein phosphorylation in regulating mycobacterial cell wall synthesis. More specifically it represents a systematic identification of novel phosphatase inhibitors that could be developed as antimicrobial agents without off-target effects on human phosphatases. Given the clinical need for new antibiotics and the possibility of combining this strategy with other antituberculosis agents, it represents a new avenue of pharmacotherapy against one of the most significant bacterial pathogens of humankind—one that kills over 3400 people each day and is evolving to be more resistant and is erasing the gains made in controlling it over the last 20 years. Moreover, this may reinvigorate the phosphatase field to search for novel inhibition methods for phosphatases implicated in a wide range of diseases such as cancer and prove that these enzymes are not as “undruggable” as once thought.

## Experimental procedures

### Chemicals and reagents

All chemicals were purchased through Fisher Scientific or Sigma-Aldrich with the following exceptions. Restriction enzymes were from New England Bio Labs. Chloramphenicol was from Life Science Products. TMED, acrylamide, and ammonium persulfate were from Biorad. GS4FF resin was from GE Healthcare. Meropenem was from APP Pharmaceuticals. ATP-Glo, DNA ladder, and glycerol were from Promega. Isoniazid was from Selleck Chemicals. GW779439X was from Med Chem Express. Antibodies and cell dyes for flow experiments were from ThermoFisher except ghost dye from Tonobo Biosciences, and pGsk3b, Capsase 3/7, and Cdk2 antibodies were from Cell Signaling. Experimental phosphatase inhibitors were from Life Chemicals. All standard reagents were of USP grade or better, and ungraded experimental reagents were of the highest purity and quality available from the supplier.

### Robotic scripting and calibration

After the high-throughput screen, all subsequent assays were conducted at the Rocky Mountain Regional Veterans Affairs Medical Center. Assays where compounds were serially diluted and plated together partially utilized a Tecan 100 liquid handling robot with EvoWare scripting software. Custom liquid classes were developed for DMSO, buffers, and detection reagents, and calibration was done colorimetrically with potassium permanganate and fluorescently with fluorescein. Protein dispensing was calibrated with BSA and Bradford reagent. Dispense distance, tip contact, and plunger speed and acceleration were calibrated until liquid handling error matched the specification limits given by the manufacturer and between-channel variation was not significant with a two-way AVOVA test (GraphPad PRISM). Shaking and temperature control were performed with an integrated Inheco plate deck control unit. All tips were Tecan electroconductive LiHa tips for liquid detection. The robot was programmed to remove and replace lids within the enclosed unit to prevent particulate contamination and maintain sterility. Liquid handling was done with this system except for the final step of adding bacteria or cells in applicable assays, which was done in a biological safety cabinet to prevent contamination of the liquid handling system.

### Strains and cloning

Recombinant PknB, GarA, and PknB K40A were constructed as described in previous work (17). PstP was cloned similarly: a sequence encoding for PstP amino acids 1 to 241 was codon optimized and ordered from IDT as a gBlock. The gBlock was digested with BamHI/NotI and ligated into Pgex-6p (GE) to express as a cleavable GST tag or PQlinkH as a cleavable HIS tag. Vectors were transformed into *E. coli* DH5a cells for protein expression. The following strains were used for microbiological experiments: *M. tuberculosis* Erdman (BSL-3) and *M. tuberculosis* H37Rv ΔRD1 ΔpanCD strain mc^2^6030 (BSL2 auxotroph).

### Protein purification

PknB and GarA were expressed and purified as described previously (17). PstP was expressed and purified similarly as follows. PstP was purified similar to previous procedures (23, 25), with the following modifications. PstP was grown at 37 °C to an OD_600_ of 0.6 to 0.8 and induced overnight with 0.5 mm IPTG at 15 °C at 200 RPM. Cells were collected and lysed in buffer A [25 mm Tris pH 8.0, 150 mm NaCl, 1 mm DTT, and 1 mm MnCl_2_] and purified by affinity chromatography. Affinity tags were digested o/n at 4 °C with 1/80 w/w PreScission protease, and further purified by anion exchange, and size exclusion chromatography following procedures for PknB, except 1 mm MnCl_2_ was substituted for MgCl_2_. The final buffer for PstP for size exclusion (and subsequent biochemical assays) was 10 mm Tris pH 7.4, 150 mm NaCl, 1 mm DTT, and 1 mm MnCl_2,_ and final purity was 95% or greater. A custom Akta Pure 25L was used for anion and size exclusion chromatography, and chromatograms were visualized and analyzed using Unicorn seven software (Cytivia).

### High-throughput screening

A biochemical assay utilizing dephosphorylation of para-nitrophenyl phosphate (pNPP) was developed for the high throughput screen. The reaction buffer used was 10 mm Tris pH 8.0, 150 mm NaCl, 1 mm DTT, and 40 mm MnCl_2_. Manganese concentrations were based on 10× the K_i_ for manganese (25) and were required for activity on reasonable time intervals. EDTA abrogated activity and was used as an inhibition control since there were no known biochemical inhibitors prior to this screen. For kinetic analysis, eight-point two-fold serial dilutions of pNPP from 16 mm to 0 mm were combined with recombinant PstP at 0.4 μm and allowed to react for 30 min at 37 °C. Product formation was measured colorimetrically at 405 nm. The data were processed in Excel (Microsoft) and non-linear regression models were fit in PRISM (GraphPad). The data fitted with a Michaelis-Menten kinetic model. EDTA at a final concentration of 10 to 0 mm was used to determine inhibition kinetics. PstP was added for a final concentration of 0.4 μm (0.01 mg/ml) and allowed to incubate for 10 min at 37 °C. Substrate was added for a final concentration of 4 mm to initialize the reaction. The reaction proceeded and was quantified as described for enzyme kinetics. The data were transformed to log scale and non-linear regression was performed in PRISM using the variable slope four-parameter model for enzyme inhibition to determine IC_50_. K_i_ values were determined using Equation 1 (17, 47).

(1) $$K_{i}=\frac{{IC}_{50}-\frac{\left[E_{t}\right]}{2}}{1+\frac{\left[S\right]}{K_{m}}}$$

Where [E_t_] is the total enzyme concentration (0.4 μm), [S] is the substrate (pNPP) concentration (4 mm), and K_m_ is the K_m_ calculated for the kinetic experiments (760 μm).

The high-throughput screen for PstP inhibitors was performed at the University of Wisconsin-Madison Carbone Cancer Center Small Molecule Screening Facility. Life Chemicals Diversity libraries 1 to 4 were chosen for the screen as they contain diverse compounds with no structure overlap and favorable drug-like properties. A total of 100,629 compounds (94,043 unique formulations and 93,948 unique base molecules) were screened. Compounds were plated from stock libraries to 2 mm in DMSO using a Beckman fX Liquid Handling robot. Compounds and controls were combined with assay buffer (10 mm Tris pH 7.4, 150 mm NaCl, 1 mm DTT, and 40 mm MnCl_2_), substrate, and protein in clear 384 well plates using a Formulatrix Tempest contact free dispenser. The final concentration of components was: 33 μm compounds, 1% DMSO, 2.5 mm pNPP, and 0.4 μm PstP. EDTA was used as a positive control at 4 mm. The reaction proceeded as above and was read on a BMG Pherastar Multimode plate reader and 405 nm. Data processing was done in CDD Vault to calculate % inhibition and z-score.

Hits were defined as any compound with greater than 20% inhibition (three standard deviations above the mean) relative to EDTA as a positive control and DMSO as a negative control. Hits meeting these criteria were selected for confirmation using the same assay with a dose response titration. Compounds were serially diluted in DMSO from 128 μm to 1 μm for an eight-point curve and subjected to the same reaction conditions as described for the screen. Data were analyzed in CDD Vault as above and % inhibition normalized to positive and negative controls. The inactive range was defined as an IC_50_ that is more than three standard deviations from the negative control mean. This dataset is available on PubChem with AID# 2060911.

### Phosphatase activity assays

A phosphatase activity assay utilizing a natural protein substrate was developed to assess PstP kinetics and small molecule inhibition in a biologically relevant system. This assay used a malachite green solution to detect free phosphate, similar to previous work on PP2A (34). PstP was purified as described above. Initial purification of the cytoplasmic domain of PknB (1-331) with a catalytically dead mutation (K40A) to minimize background activity was performed as previously described (17). After digested GST was separated by anion exchange chromatography, PknB K40A was phosphorylated for 2 hours at 37 °C by a GST tagged WT PknB cytoplasmic domain (purified as above) at a ratio of 1/10 w/w with a 10× molar ratio of ATP and 10 mm MgCl_2_ in the buffer from the eluting fractions on anion exchange (20 mm Tris pH 8.0, 1 mm DTT, and approximately 150 mm NaCl). After phosphorylation, active GST-PknB was removed by passing three times over GS4FF columns, and columns were rinsed with matched buffer. Phospho-PknB was concentrated and injected on size exclusion chromatography (Sepharose 6, Cytivia, packed in a 10/300 Tricorn column) to separate pPknB from ATP and exchange to a uniform storage buffer to match the assay buffer (10 mm Tris 7.4, 150 mm NaCl, 1 mm DTT). Protein was concentrated, flash frozen in liquid nitrogen, and stored at −80 °C. Purity was at least 95% as visualized on SDS-PAGE. Prior to use, protein was slow thawed on ice, centrifuged to remove precipitates (if any), and concentration was determined by Bradford assay. Fresh assay buffer (10 mm Tris 7.4, 150 mm NaCl, 1 mm DTT, 4 mm MnCl_2_) was made for each assay. All assays were performed in duplicate at least three times.

For the kinetic assay, pPknB was serially diluted at a 0.6× ratio for a target final concentration gradient starting at 180 μm. A matched substrate curve with buffer instead of enzyme was used to correct for substrate background. PstP was added for a final concentration of 500 nm and a final volume of 50 μl and allowed to react at 37 °C for 20 min. The assay was stopped, and protein precipitated, by the addition of 5 μl of 6M HCl. Protein was allowed to precipitate for 2 min then the plate was spun for 5 min at 4000 RPM. Forty-5 μl of clarified reaction mixture was removed from the plate and was partially neutralized by adding 4 μl of 6M NaOH. After neutralization for 2 min, 100 μl of malachite green phosphate detection solution (35) was added, and the plate was visualized for uniform starting color. Wells with immediate olive-green color change (Mn oxidation state change from incomplete mixing) were excluded but at no more than two per plate. Chromophore formation was quantified at OD_620_ after a 10-min incubation at room temperature. KH_2_PO_4_ was used as a standard to determine the generated phosphate and the reaction velocity was calculated and graphed against concentration. To determine enzyme kinetics, nonlinear regression analysis was performed with GraphPad PRISM, and Michaelis-Menten and allosteric sigmoidal models were fit and compared.

For PstP inhibition assays, 37.5 μl of PstP (target final concentration of 500 nm) was mixed with 1 μl of compounds in DMSO, which were serially diluted from 10 mm to 0 (control) at a factor of 0.6× for final concentration of 200 μm to 0. Compounds and the enzyme were incubated at room temperature for 10 min. Phospho-PknB was added to a final concentration of 40 μm (k_1/2_) and a final volume of 50 μl. The reaction proceeded and was quantified as for kinetics, with the exception that a filter plate was used to separate precipitated protein due to DMSO preventing compact pelleting. IC_50_ was determined with GraphPad PRISM using a non-linear variable slope 4-parameter model, and k_i_ was calculated from IC_50_ using Equation 1.

### Docking

Computational docking was done as follows: The LifeChem pre-plated diversity library SMILES were processed with OpenEye ligand preparation tools (Cadence Molecular Sciences). Fixpka (QUACPAC v2.1.2.1) was used to protonate (pH 7.4). Omega v5.0.0.3 was used to enumerate ring conformers, ambiguous stereocenters, and build 3D structures in low energy conformations for docking (48). MMFF partial charges were assigned using the molcharge utility (QUACPAC v2.1.2.1). Target protein PstP (PDB ID: 1TXO) was prepared for docking using OpenEye’s SPRUCE utility (v1.3.0.1) and docked with OpenEye’s FRED (v4.1.0.1) (39). Poses were rendered using PyMOL (v3.1.6.1, Schrodinger, Inc.).

### Microbiologic assays

Three assays were utilized to assess microbiologic inhibition. The resazurin assay utilizing a BSL2 auxotroph (mc^2^6030) was used to do initial screening and checkerboard assays. A colony-forming unit (CFU) assay was used with the same strain to confirm key conditions gleaned from the resazurin assays and assess bactericidal or bacteriostatic behavior. Finally, a CFU/OD_600_ assay was used in the BSL3 suite with the *Mtb* Erdman strain to test select conditions against a pathogenic strain. Resazurin assays generated copious numbers of growth curves, and for clarity, IC_50_/IC_90_ data are presented in main text Figures 1, 3, and 4, with all growth curves shown in Supporting Information (SI) Figures S2, S4, and S5. Erdman data was collected over an 8-day time course. This data is presented in Supporting Information Figure S6 and is analyzed and summarized in main text Figure 5. In-text callouts cite the main figure and associated Supporting Information curves.

### Resazurin assay

The resazurin assay was performed analogously to previous work (17), with some changes. The liquid media was 7H9 +OADC with the addition of 0.5% glycerol, 0.2% casamino acids, 24 μg/ml pantothenate, and 0.05% tyloxapol. Solid media was 7H11 with the same addition of OADC, casamino acids, and pantothenate. *Mtb* (mc^2^6030) was streaked from frozen culture and grown on solid media for 1 month until colonies appeared. Colonies were selected and grown in liquid media for an additional 2 to 4 weeks until reaching an OD_600_ between 0.100 and 0.300. One-milliliter aliquots were portioned and frozen for subsequent use. For experiments, an aliquot was placed in 25 ml of liquid media and grown for 3 days at 37 °C at 200 RPM. Bacteria were carefully adjusted to an OD_600_ of 0.100 ± 0.005 and then diluted by one-tenth (final OD_600_ of 0.010).

For simple minimum inhibitor concentration (IC_90_) assays, inhibitor plates were created using the liquid handling robot. Inhibitors in DMSO were placed in the first column of the center 60 wells of a skirted hardshell PCR plate (Biorad) at 100 times the final concentration desired. Inhibitors were plated at 20 mm for a final concentration of 200 μm or at their solubility maximum. The robot distributed DMSO to the remaining center wells and serially diluted the inhibitors from the first column using two-fold dilution and 10× mixing to create a nine-point curve with the last column remaining as DMSO for controls. Two hundred μl of sterile water was distributed in the outside wells of a sterile black clear bottom 96 well plate and 98 μl of media was then added to the center 60 wells. Two μl of serially diluted inhibitors was copied from the inhibitor plate to the black plate and mixed. The plate was removed from the robot after assembly and 100 μl of diluted bacteria was added to each well, bringing the final starting OD_600_ to 0.005 and DMSO concentration to 1% with inhibitors from 200 μm to 0. The plate was incubated stationary for 5 days in a humidified incubator at 5% CO_2_. Twenty microliter of sterile filtered resazurin stock solution (1 mg/ml in water) were added to the center wells and to six of the outer water wells (adjusted to 200 μl) to be used as blanks. The plate was incubated for 3 days and fluorescence was read on a Promega GloMax with 520 nm excitation and 580 to 640 nm emission filters. Baseline was computed using blanks and fluorescence units were converted to percent growth using an average of the DMSO only controls. IC_50_ and IC_90_ were determined by fitting a nonlinear regression curve in GraphPad PRISM using the normalized untransformed hill model with variable slope. IC_50_ and IC_90_ were constrained to be between 0 and the highest concentration tested (200 μm) and hillslope was constrained to have an absolute value between 0 and 4.

For checkerboard assays, the maximum concentration of each compound was set at or near their respective IC_90_s. PstP inhibitors in DMSO at 100× were placed in a column and then serially diluted with the robot so that each row was a copy of the other. Two inhibitors were used per plate in the center wells only, with the last column of each being DMSO. The compound to be tested against the PstP inhibitors was serially diluted in media and 98 μl of media with compound was distributed across a black plate as above. Two μl of PstP inhibitors from the plate were copied and mixed, and bacteria were added as described above. Plates were then incubated and read as described above as well. If a third compound was being assessed in a “3D checkerboard” format, this compound was added to all the media prior to the second compound being serially diluted. This ensured that each well contained the same sub-lethal dose of the third compound. IC_50_ was used for comparative data since nonlinear regression was more robust for this central value as opposed to the IC_90_ which is the highest concentration tested and regression carries more error at the ends of the curve.

Fractional inhibitory concentration (FIC) was calculated and assessed based on previous work (17, 49). IC_90_ was used for FIC calculations to match historical data and other published definitions. FICs in experiments with a simple 2D checkerboard were calculated with Equation 2 and those with a 3D checkerboard were calculated with Equation 3.

(2) $$FIC=\frac{C_{A}\;}{{MIC}_{A}}+\frac{C_{B}\;}{{MIC}_{B}}$$

(3) $$FIC=\frac{C_{A}\;}{{MIC}_{A}}+\frac{C_{B}\;}{{MIC}_{B}}+\frac{C_{C}\;}{{MIC}_{C}}$$

Where C_A_ is the concentration of compound A that is effective at a given concentration of compound B (C_B_) or in combination with another, C_C_, and MIC_X_ is the IC_90_ of each compound alone.

Equation 3 is an extrapolation of Equation 2, commonly used (49), and others have extrapolated this similarly (33). FIC index values between 0.5 and 2.0 are mathematically additive and below this range synergistic and above antagonistic (49). These ranges were established concerning two inhibitors only, but since some assays are utilizing three inhibitors, we calculate that additive effects are centered around an FIC of 1.5 instead of 1.0 and thus synergy is considered below an FIC of 0.75 and antagonism above 3.0 for 3D experiments.

### CFU assay (BSL2)

Bacterial viability after treatment was assessed with a colony-forming unit (CFU) assay. Concentrations centered around the area of dynamic changes (one quarter of the IC_90_ of each compound) and the IC_90_s individually were chosen to test. Media and compounds at desired concentrations or DMSO were added to 96-well plates, in duplicate, with the liquid handling robot as described for the resazurin assays. *Mtb* (mc^2^6030) was added as described as well, with a final inoculum OD_600_ of 0.005, which corresponds to an average of 45,600 ± 3350 CFU/ml. Plates were grown for 5 days as above and after, 100 μl from each well was serially diluted to 1/128 or 1/256 and plated on 7H11 media with OADC, 0.2% casamino acids, and 24 μg/ml pantothenate with no antibiotics or inhibitor treatment. Bacteria grew on the plates for 10 days. Lower dilutions or longer timepoints resulted in lawns in the no treatment controls. Colonies were counted using an IncuCount Automatic Colony Counter 150 (RevSci) with the following parameters: focus: 138, exposure: 55, brightness: 70, contrast: 80. A colony size profile was set by standardizing to a plate of WT bacteria of a known count. To normalize the CFU data based on inoculum, specific growth rate (SGR) was calculated based on Equation 4 (50). The IC_90_ was defined as less than 10% growth and the minimum bactericidal concentration (MBC) was defined as the dose that killed 99.9% of the initial inoculum. Since only two combinatorial treatments at less than IC_90_ were used for CFU assays, an FIC could only be calculated from the lowest combined effective treatment and is denoted as FIC_A_ for each group.

(4) $$SGR=\frac{\ln\left({CFU}_{tn}\right)-\ln\left({CFU}_{t0}\right)}{\left(t_{n}-t_{0}\right)}$$

Where t_n_ is the treatment time with the bacteria (120 h) and t_0_ is time zero and the CFU counts are those corresponding to that at the respective time points.

### Pathogenic (BSL3) tuberculosis CFU assay

Cultures of virulent *Mtb* Erdman were propagated in Middlebrook 7H9 medium containing ADS supplement and 0.05% tween80 (51, 52). Mid-log *Mtb* cultures were diluted to an OD_600_ = 0.05, and 5 ml aliquots were inoculated into sterile glass test tubes (20 by 125 mm) containing a 12 × 4.5 mm stir bar. Cultures were incubated at 37 °C in 5% CO_2_ while stirring at ∼200 rpm under the control of a rotary magnetic tumble stirrer (53). Following 18 h outgrowth, drugs were added from sterile 200X stocks prepared in DMSO at indicated concentrations. Drug-free vehicle control cultures were supplemented with DMSO at a final concentration of 0.5%. Drug-exposed cultures were incubated for 8 days with CFU assessments at days 0, 1, 2, 4, and eight post drug exposure. SGR was calculated with Equation 4 as defined above, except that the treatment time used (t_n_) was 96 h. IC_90_ and MBC are defined as in the BSL2 CFU assay.

### Cytotoxicity assays

Cytotoxicity was measured for microbiologically active compounds using a two-panel approach. Panel A utilized surface staining and consisted of Ghost Dye (Tonobo) to measure viability, and Annexin V (Biolegend) and Caspase 3/7 dye (ThermoFisher) to assess apoptotic stress. Panel B utilized intracellular markers and consisted of DAPI (Biolegend), and antibodies to Cdk2 (PE, Cell Signaling), Akt1 pS473 (e450, ThermoFisher), mTor pS2448 (PE-Cy7, ThermoFisher), p38 MAPK alpha pT180, pY182 (FITC, ThermoFisher), and Gsk3β pS9 (AF647, Cell Signaling). THP-1 cells were purchased through ATCC and grown following standard methods (40).

### Inhibitor incubation

For panel A, compounds were serially diluted two-fold in DMSO from 20 mm to 0 mm in 10-point curves in the center of a 96-well plate. Two hundred microliter of sterile water was plated to the outside wells of a 96-well plate, and 2 μl of diluted compounds were added to 98 μl of THP-1 media in the center wells. Cells were spun at 400*g* for 5 min to pellet and resuspended at approximately 5 × 10^6^ cells/ml. Hundred microliter of cells were added to each well for a final cell count of approximately 250,000/well. Each plate received the same amount of cells/well and plate-to-plate counts only varied ± 50,000 cells/well. Plates grew at 37 °C 5% CO_2_ for 3 days prior to harvest. Three concentrations for panel B were chosen based on the CC_50_ determined in panel A. Compounds were plated at a tested integer closest to the CC_50_, double that concentration and 1/10 of that doubled concentration. For example, a CC_50_ of 46.7 μm would be plated at 50 μm, 100 μm, and 10 μm. These concentrations corresponded closely with the center and shoulders of the CC_50_ curve.

### Staining: panel A

A 10X Annexin V Buffer was prepared by combining 100 mm HEPES (pH 7.4), 1400 mm NaCl, and 25 mm CaCl_2_. To prepare a 1X working solution, 10 ml of the 10X buffer was diluted in 90 ml molecular-grade water. Antibodies were retrieved from appropriate storage: Caspase and 2X Live/Dead antibodies from −30 °C and Annexin V antibody from 4 °C. All antibodies were kept on ice prior to use.

Cells were harvested by centrifuging at 600*g* for 10 min and aspirating the media from each well, followed by two washes with 250 μl 1X Annexin V Buffer per well. For each wash, the buffer was added, gently mixed, and aspirated completely after centrifugation. A 1X Live/Dead antibody solution was prepared by combining 35 μl of 1X Annexin V Buffer with 35 μl of 2X Live/Dead antibody. The solution was mixed thoroughly by vortexing. Cells were transferred to a V-bottom 96-well plate by adding 100 μl 1X Annexin V Buffer to each well, mixing by pipetting, and aspirating the entire well volume into the new plate. The cells were then centrifuged at 600*g* for 10 min, and the supernatant was aspirated carefully to avoid disturbing the pellet. The cell pellet was resuspended in 250 μl 1X Annexin V Buffer for a subsequent wash.

A Master Mix for staining was prepared by combining 155 μl Caspase antibody, 155 μl Annexin V antibody, and 62 μl 1X Live/Dead solution. Following centrifugation (600*g*, 10 min) and supernatant removal, cells were resuspended in 50 μl 1X Annexin V Buffer. Subsequently, 6 μl of the Master Mix was added to each sample well, excluding fluorescence-minus-one (FMO) control wells. FMO controls were prepared using the following antibody combinations: Caspase FMO: 2.5 μl Annexin V antibody and 1 μl 1X Live/Dead antibody. Annexin V FMO: 1 μl Caspase antibody and 1 μl 1X Live/Dead antibody. Live/Dead FMO: 2.5 μl Annexin V antibody and 1 μl Caspase antibody. After antibody addition, the plate was gently vortexed (power ∼6–7) and incubated at 4 °C for 45 min. Cells were then washed by adding 200 μl 1X Annexin V Buffer, centrifuged (600*g*, 10 min), and the supernatant was discarded. Fixation was performed by resuspending the cells in 2% paraformaldehyde (PFA), prepared by diluting 4% PFA in DPBS, and incubating for 30 min.

### Staining: panel B

THP-1 cells were plated at a density of 2.5 × 10^5^ cells/well in 96-well plates containing novel phosphatase inhibitors and vehicle controls, then incubated for 72 h. After incubation, the cells were harvested, transferred to a V-bottom 96-well plate, and washed twice with 1X FACS buffer (Dulbecco’s Phosphate Buffered Saline (D-PBS) with 1% (w/v) BSA, 636.463 μm EDTA, and 2 mm sodium azide). All centrifugation steps were performed at 600*g* for 10 min. The cells were then fixed in 100 μl of 2% paraformaldehyde in 1X FACS buffer for 30 min at room temperature.

The cells were stained for 1 h at 4 °C in 50 μl of Permeabilization Medium B (GAS002S5; Invitrogen) with the following antibodies and dye: 2.5 μl phospho-p38 MAPKα (T180, Y182) recombinant rabbit monoclonal antibody FITC (MA5-37230, Lot: YI4040421; ThermoFisher), 2.5 μl phospho-mTOR (S2448) mouse monoclonal antibody PerCP-eFluor 710 (46-9718-42, ThermoFisher), 1 μl phospho-GSK-3β (S9) (D85E12) recombinant rabbit monoclonal antibody AF 647 conjugate (144,332, Lot: 5; Cell Signaling Technology), 1 μl CDK2 (78B2) rabbit monoclonal antibody PE conjugate (14174S, Lot: 3; Cell Signaling Technology), 2.5 μl phospho-AKT1 (S473) monoclonal antibody PE-Cyanine7 (25-9715-42, Lot: 2473758; ThermoFisher), and 5 μl 4′,6-diamidino-2-phenylindole dilactate (DAPI; D3571, Invitrogen). After staining, the cells were washed twice, fixed as described above, and then transferred to 5 ml culture tubes.

### Flow cytometry

Panel A samples were analyzed using the Cytoflex (BD Biosciences). Samples for Panel B were analyzed using the BD FACSymphony A5 SE Cell Analyzer.

### Data analysis

Flow analysis was performed using Flow Jo version 10.10 (Ashland, OR). Panel A: Cells were gated as follows: FSC vs SSC, two singlet gates to discriminate doublets, gating on live/dead cells, and gating on annexin V and caspase positive or negative cells (Fig. S7). Panel B: Cells were gated as follows: FSC vs SSC, two singlet gates to discriminate doublets, DAPI stain to determine the cell cycles, and SSC low determined by gating on 90% of untreated cells to eliminate more granulated cells (Fig. S9, *A*–*E*). Each intracellular marker was examined for MFI in the DAPI+, G0/G1 phase, S phase and M/G2 phase (Fig. S9*F*). Samples with lower than 70% DAPI + staining and for which we were unable to determine cell cycle phases as well as outliers that were identified at Q = 1% in GraphPad PRISM were excluded from analysis.

## Data availability

All data needed to evaluate the conclusions in the paper are present in the paper and/or the Supplementary Materials. High Throughput Screening (HTS) and confirmatory screen data are deposited in PubChem and can be found at AID# 2060911. Materials, such as constructs, can be provided by N. Wlodarchak pending scientific review and a completed material transfer agreement. Requests should be submitted to the corresponding author.

## Supporting information

This article contains supporting information.

## Conflict of interest

The authors declare the following financial interests/personal relationships which may be considered as potential competing interests: N. Wlodarchak is an inventor on U.S. patent #9540369 “Use of kinase inhibitors to increase the susceptibility of Gram + bacteria to b-lactam antibiotics” and U. S. patent # 62972349 “Inhibitors of Bacterial PASTA Kinases”.

## References

[1] (2024). Global Tuberculosis Report 2024.

[2] Williams P.M., Pratt R.H., Walker W.L., Price S.F., Stewart R.J., Feng P.I. (2024). Tuberculosis — United States. MMWR Morb. Mortal. Wkly. Rep..

[3] Seung K.J., Keshavjee S., Rich M.L. (2015). Multidrug-resistant tuberculosis and extensively drug-resistant tuberculosis. Cold Spring Harbor Perspect. Med..

[4] Grant S.K. (2009). Therapeutic protein kinase inhibitors. Cell Mol. Life Sci..

[5] Cohen P. (2002). Protein kinases--the major drug targets of the twenty-first century?. Nat. Rev. Drug Discov..

[6] Roskoski R. (2020). Properties of FDA-approved small molecule protein kinase inhibitors: a 2020 update. Pharmacol. Res..

[7] Chawla Y., Upadhyay S., Khan S., Nagarajan S.N., Forti F., Nandicoori V.K. (2014). Protein kinase B (PknB) of Mycobacterium tuberculosis is essential for growth of the pathogen in vitro as well as for survival within the host. J. Biol. Chem..

[8] Narayan A., Sachdeva P., Sharma K., Saini A.K., Tyagi A.K., Singh Y. (2007). Serine threonine protein kinases of mycobacterial genus: phylogeny to function. Physiol. genomics.

[9] Jones G., Dyson P. (2006). Evolution of transmembrane protein kinases implicated in coordinating remodeling of gram-positive peptidoglycan: inside versus outside. J. Bacteriol..

[10] Kang C.M., Abbott D.W., Park S.T., Dascher C.C., Cantley L.C., Husson R.N. (2005). The Mycobacterium tuberculosis serine/threonine kinases PknA and PknB: substrate identification and regulation of cell shape. Genes Dev..

[11] Wright D.P., Ulijasz A.T. (2014). Regulation of transcription by eukaryotic-like serine-threonine kinases and phosphatases in Gram-positive bacterial pathogens. Virulence.

[12] Pensinger D., Schaenzer A., Sauer J.-D. (2017). Do shoot the messenger: PASTA kinases as virulence determinants and antibiotic targets. Trends Microbiol..

[13] Chapman T.M., Bouloc N., Buxton R.S., Chugh J., Lougheed K.E., Osborne S.A. (2012). Substituted aminopyrimidine protein kinase B (PknB) inhibitors show activity against Mycobacterium tuberculosis. Bioorg. Med. Chem. Lett..

[14] Naqvi A., Malasoni R., Srivastava A., Pandey R.R., Dwivedi A.K. (2014). Design, synthesis and molecular docking of substituted 3-hydrazinyl-3-oxo-propanamides as anti-tubercular agents. Bioorg. Med. Chem. Lett..

[15] Sipos A., Pato J., Szekely R., Hartkoorn R.C., Kekesi L., Orfi L. (2015). Lead selection and characterization of antitubercular compounds using the nested chemical library. Tuberculosis (Edinburgh, Scotland).

[16] Wang T., Bemis G., Hanzelka B., Zuccola H., Wynn M., Moody C.S. (2017). Mtb PKNA/PKNB dual inhibition provides selectivity advantages for inhibitor design to minimize host kinase interactions. ACS Med. Chem. Lett..

[17] Wlodarchak N., Teachout N., Beczkiewicz J., Procknow R., Schaenzer A.J., Satyshur K. (2018). In silico screen and structural analysis identifies bacterial kinase inhibitors which act with beta-lactams to inhibit mycobacterial growth. Mol. Pharm..

[18] Wlodarchak N., Feltenberger J.B., Ye Z., Beczkiewicz J., Procknow R., Yan G. (2021). Engineering selectivity for reduced toxicity of bacterial kinase inhibitors using structure-guided medicinal chemistry. ACS Med. Chem. Lett..

[19] Smith T., Wolff K.A., Nguyen L. (2013). Molecular biology of drug resistance in Mycobacterium tuberculosis. Curr. Top. Microbiol. Immunol..

[20] Debarbouille M., Dramsi S., Dussurget O., Nahori M.A., Vaganay E., Jouvion G. (2009). Characterization of a serine/threonine kinase involved in virulence of Staphylococcus aureus. J. Bacteriol..

[21] Tamber S., Schwartzman J., Cheung A.L. (2010). Role of PknB kinase in antibiotic resistance and virulence in community-acquired methicillin-resistant Staphylococcus aureus strain USA300. Infect. Immun..

[22] Schaenzer A.J., Wlodarchak N., Drewry D.H., Zuercher W.J., Rose W.E., Ferrer C.A. (2018). GW779439X and its pyrazolopyridazine derivatives inhibit the serine/threonine kinase Stk1 and act as antibiotic adjuvants against beta-lactam-resistant Staphylococcus aureus. ACS Infect. Dis..

[23] Boitel B., Ortiz-Lombardia M., Duran R., Pompeo F., Cole S.T., Cervenansky C. (2003). PknB kinase activity is regulated by phosphorylation in two Thr residues and dephosphorylation by PstP, the cognate phospho-Ser/Thr phosphatase, in Mycobacterium tuberculosis. Mol. Microbiol..

[24] Sharma A.K., Arora D., Singh L.K., Gangwal A., Sajid A., Molle V. (2016). Serine/threonine protein phosphatase PstP of Mycobacterium tuberculosis is necessary for accurate cell division and survival of pathogen. J. Biol. Chem..

[25] Pullen K.E., Ng H.L., Sung P.Y., Good M.C., Smith S.M., Alber T. (2004). An alternate conformation and a third metal in PstP/Ppp, the M. tuberculosis PP2C-Family Ser/Thr protein phosphatase. Structure.

[26] Burnside K., Lembo A., de Los Reyes M., Iliuk A., Binhtran N.T., Connelly J.E. (2010). Regulation of hemolysin expression and virulence of Staphylococcus aureus by a serine/threonine kinase and phosphatase. PLoS One.

[27] Chatterjee A., Poon R., Chatterjee S.S. (2020). Stp1 loss of function promotes β-Lactam resistance in Staphylococcus aureus that is independent of classical genes. Antimicrob. Agents Chemother..

[28] Beltramini A.M., Mukhopadhyay C.D., Pancholi V. (2009). Modulation of cell wall structure and antimicrobial susceptibility by a Staphylococcus aureus eukaryote-like serine/threonine kinase and phosphatase. Infect. Immun..

[29] Nahid P., Pai M., Hopewell P.C. (2006). Advances in the diagnosis and treatment of tuberculosis. Proc. Am. Thorac. Soc..

[30] Caminero J.A., Sotgiu G., Zumla A., Migliori G.B. (2010). Best drug treatment for multidrug-resistant and extensively drug-resistant tuberculosis. Lancet Infect. Dis..

[31] Vainonen J.P., Momeny M., Westermarck J. (2021). Druggable cancer phosphatases. Sci. Transl. Med..

[32] Zheng W., Cai X., Xie M., Liang Y., Wang T., Li Z. (2016). Structure-based identification of a potent inhibitor targeting Stp1-Mediated virulence regulation in Staphylococcus aureus. Cell Chem. Biol..

[33] Hamoud R., Reichling J., Wink M. (2015). Synergistic antibacterial activity of the combination of the alkaloid sanguinarine with EDTA and the antibiotic streptomycin against multidrug resistant bacteria. J. Pharm. Pharmacol..

[34] Wlodarchak N., Guo F., Satyshur K.A., Jiang L., Jeffrey P.D., Sun T. (2013). Structure of the Ca2+-dependent PP2A heterotrimer and insights into Cdc6 dephosphorylation. Cell Res..

[35] Harder K.W., Owen P., Wong L.K., Aebersold R., Clark-Lewis I., Jirik F.R. (1994). Characterization and kinetic analysis of the intracellular domain of Human Protein Tyrosine Phosphatase beta (HPTP beta) using synthetic phosphopeptides. Biochem. J..

[36] Shamma F., Rego E.H., Boutte C.C. (2022). Mycobacterial serine/threonine phosphatase PstP is phosphoregulated and localized to mediate control of cell wall metabolism. Mol. Microbiol..

[37] Duran R., Villarino A., Bellinzoni M., Wehenkel A., Fernandez P., Boitel B. (2005). Conserved autophosphorylation pattern in activation loops and juxtamembrane regions of Mycobacterium tuberculosis Ser/Thr protein kinases. Biochem. Biophys. Res. Commun..

[38] Pan C., Tang J.Y., Xu Y.F., Xiao P., Liu H.D., Wang H.A. (2015). The catalytic role of the M2 metal ion in PP2Cα. Sci. Rep..

[39] McGann M. (2011). FRED pose prediction and virtual screening accuracy. J. Chem. Inf. Model..

[40] Pick N., Cameron S., Arad D., Av-Gay Y. (2004). Screening of compounds toxicity against human monocytic cell line-THP-1 by flow cytometry. Biol. Proced. Online.

[41] Takekawa M., Maeda T., Saito H. (1998). Protein phosphatase 2Calpha inhibits the human stress-responsive p38 and JNK MAPK pathways. EMBO J..

[42] Wlodarchak N., Xing Y. (2016). PP2A as a master regulator of the cell cycle. Crit. Rev. Biochem. Mol. Biol..

[43] Liang Yi C., Sun Z., Lu C., Lupien A., Xu Z., Berton S. (2024). Discovery of benzo[c]phenanthridine derivatives with potent activity against multidrug-resistant Mycobacterium tuberculosis. Microbiol. Spectr..

[44] Su J., Schlicker C., Forchhammer K. (2011). A third metal is required for catalytic activity of the signal-transducing protein phosphatase M tPphA. J. Biol. Chem..

[45] Shamma F., Papavinasasundaram K., Quintanilla S.Y., Bandekar A., Sassetti C., Boutte C.C. (2021). Phosphorylation on PstP regulates cell wall metabolism and antibiotic tolerance in Mycobacterium smegmatis. J. Bacteriol..

[46] Lougheed K.E., Osborne S.A., Saxty B., Whalley D., Chapman T., Bouloc N. (2011). Effective inhibitors of the essential kinase PknB and their potential as anti-mycobacterial agents. Tuberculosis (Edinburgh, Scotland).

[47] Heerding D.A., Rhodes N., Leber J.D., Clark T.J., Keenan R.M., Lafrance L.V. (2008). Identification of 4-(2-(4-amino-1,2,5-oxadiazol-3-yl)-1-ethyl-7-{[(3S)-3-piperidinylmethyl]oxy}-1H- imidazo[4,5-c]pyridin-4-yl)-2-methyl-3-butyn-2-ol (GSK690693), a novel inhibitor of AKT kinase. J. Med. Chem..

[48] Hawkins P.C., Skillman A.G., Warren G.L., Ellingson B.A., Stahl M.T. (2010). Conformer generation with OMEGA: algorithm and validation using high quality structures from the protein databank and Cambridge structural database. J. Chem. Inf. Model..

[49] Meletiadis J., Pournaras S., Roilides E., Walsh T.J. (2010). Defining fractional inhibitory concentration index cutoffs for additive interactions based on self-drug additive combinations, monte carlo simulation analysis, and in vitro-in vivo correlation data for antifungal drug combinations against Aspergillus fumigatus. Antimicrob. Agents Chemother..

[50] Zwietering M.H., Jongenburger I., Rombouts F.M., van 't Riet K. (1990). Modeling of the bacterial growth curve. Appl. Environ. Microbiol..

[51] Walter N.D., Born S.E.M., Robertson G.T., Reichlen M., Dide-Agossou C., Ektnitphong V.A. (2021). Mycobacterium tuberculosis precursor rRNA as a measure of treatment-shortening activity of drugs and regimens. Nat. Commun..

[52] Reichlen M.J., Born S.E.M., Lyons M.A., Rossmassler K., Reid J., Robertson G.T. (2023). Standardized RS ratio metrics to assess tuberculosis antimicrobial efficacy and potency. Antimicrob. Agents Chemother..

[53] Leistikow R.L., Morton R.A., Bartek I.L., Frimpong I., Wagner K., Voskuil M.I. (2010). The Mycobacterium tuberculosis DosR regulon assists in metabolic homeostasis and enables rapid recovery from nonrespiring dormancy. J. Bacteriol..

