# Supplementary material for: Identification and characterization of inhibitors of the tuberculosis phosphatase PstP
Source: J Biol Chem. 2026 Feb 26;302(4):111316. doi: 10.1016/j.jbc.2026.111316 (PMC13129536; doi:10.1016/j.jbc.2026.111316)
Supplement: Supporting Information [file mmc1.pdf]

Identification and characterization of inhibitors of the tuberculosis phosphatase PstP

Chase Riedel[1], Jeremy Rahkola[1], Matthew Reichlen[2,3], Hunter Ries[4], Spencer S Ericksen[4], Anthony Gitter[5,6], Martin Voskuil[2,3], Nathan Wlodarchak[1,2,*], Nathan.Wlodarchak@ucdenver.edu

[1]Research Service, Rocky Mountain Regional Veterans Affairs Medical Center, Aurora, Colorado, USA
[2]Department of Immunology and Microbiology, University of Colorado Denver, Denver, Colorado, USA
[3]Consortium for Applied Microbial Metrics, Aurora, Colorado, USA
[4]Small Molecule Screening Facility, University of Wisconsin-Madison, Madison, Wisconsin, USA
[5]Morgridge Institute for Research, Madison, Wisconsin, USA
[6]Department of Biostatistics and Medical Informatics, University of Wisconsin-Madison, Madison, Wisconsin, USA

*For correspondence: Nathan Wlodarchak

Table of Contents

I. **Cover Page**
   A. **Title, authors, affiliations, TOC**
   B. **Excel table supplement caption**
II. **Supplemental figures & captions**
   A. **Figure S1:** ATA inhibition of PstP and Stp1
   B. **Figure S2:** *M. tuberculosis* $mc^2$6030 inhibition
   C. **Figure S3:** PstP inhibition with pPknB substrate
   D. **Figure S4:** *M. tuberculosis* $mc^2$6030 checkerboard assay INH & MEM
   E. **Figure S5:** *M. tuberculosis* $mc^2$6030 checkerboard assay GW & GW+MEM
   F. **Figure S6:** Pathogenic *M. tuberculosis* (Erdman) assays
   G. **Figure S7:** Flow cytometry gating panel A (cytotoxicity)
   H. **Figure S8:** Flow cytometry analysis panel B (PstP inhibitors on cell cycle regulation)
   I. **Figure S9:** Flow cytometry gating panel B (cell cycle regulation)
III. **Table S1** (separate file in .xlsx format)

Table S1: Results of the secondary biochemical screen including compound identification, percent inhibition, IC_{50}, curve class, and fit. IC_{90} from the microbiological screen of the 40 top biochemical performers is also shown. Additional data for microbiologically active compounds (indicated with their respective colors) is given in **Figure1**.

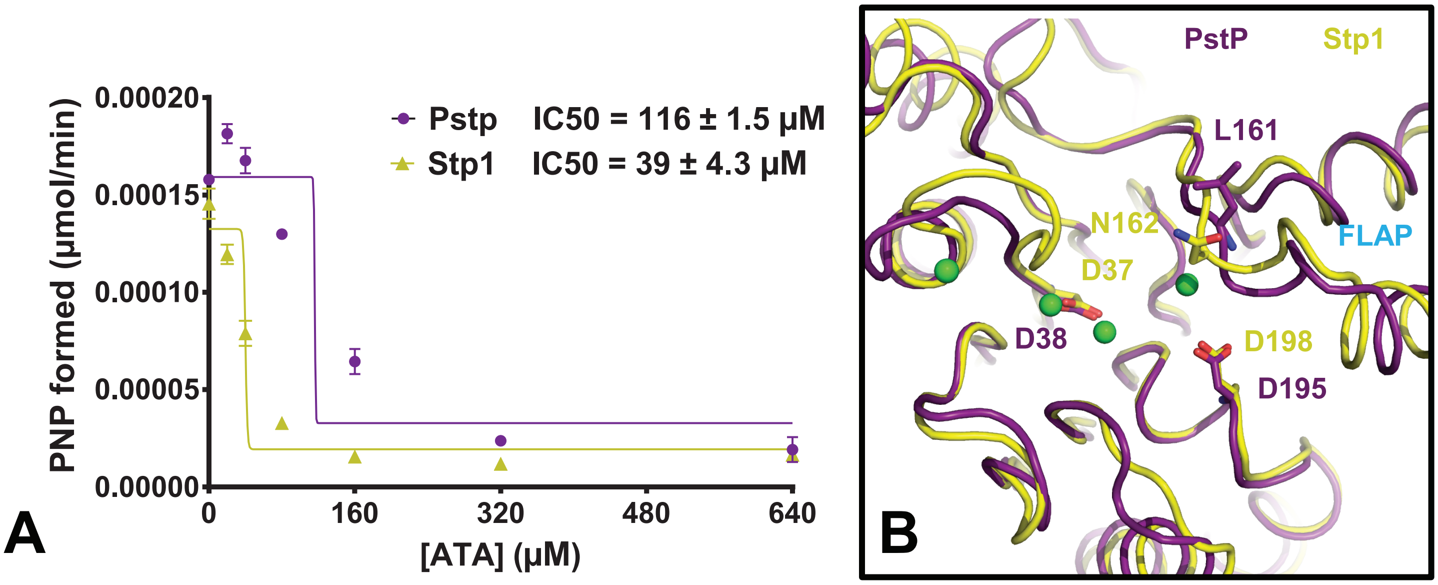

Figure S1: Aurintricarboxylic acid (ATA) poorly inhibits PstP compared to Stp1. **A**: Inhibition kinetics with pNPP for both phosphatases were done as described in the methods. IC_{50} values are given with each curve along with the structure of ATA and error bars indicate standard deviation. ATA inhibits Stp1 nearly three times more efficiently than PstP. **B**: Alignment of the crystal structures of PstP (1TXO, purple) and Stp1 (5F1M, yellow) reveal a conserved active site with a variable flap domain. Residues in Stp1 predicted to bind to ATA are shown in yellow sticks and their corresponding residues for PstP are shown in purple sticks. Metal ion locations for Stp1 are shown in green sphere and the flap is labeled in cyan. GraphPad PRISM was used to fit the data with non-linear regression (4-parameter variable slope) and calculate IC_{50}. Small molecules (**A**) are rendered with ChemDraw® and proteins (**B**) with Pymol.

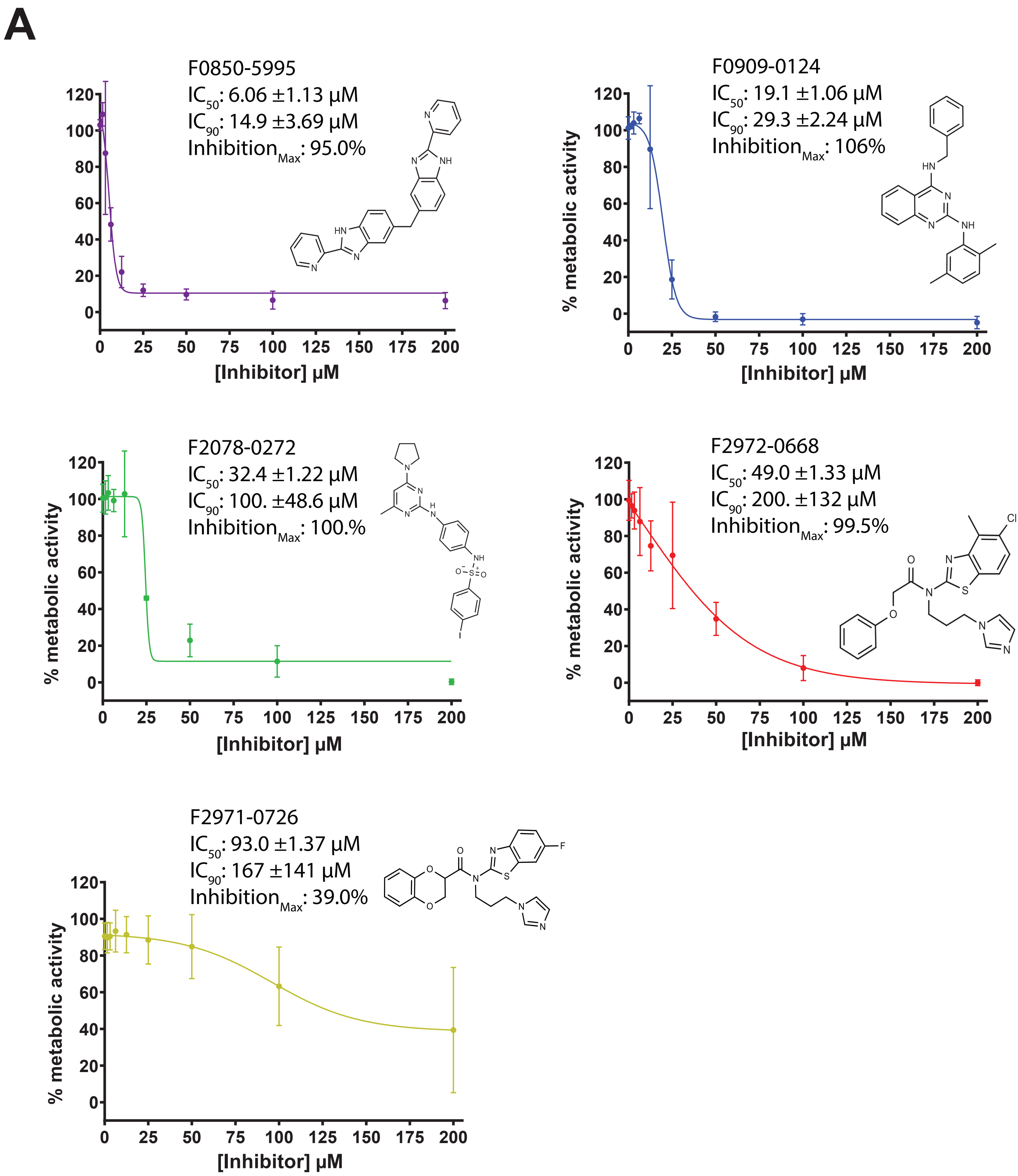
A
F0850-5995
IC50: 6.06 ±1.13 µM
IC90: 14.9 ±3.69 µM
InhibitionMax: 95.0%
F0909-0124
IC50: 19.1 ±1.06 µM
IC90: 29.3 ±2.24 µM
InhibitionMax: 106%
F2078-0272
IC50: 32.4 ±1.22 µM
IC90: 100. ±48.6 µM
InhibitionMax: 100.%
F2972-0668
IC50: 49.0 ±1.33 µM
IC90: 200. ±132 µM
InhibitionMax: 99.5%
F2971-0726
IC50: 93.0 ±1.37 µM
IC90: 167 ±141 µM
InhibitionMax: 39.0%
% metabolic activity
[Inhibitor] µM

B

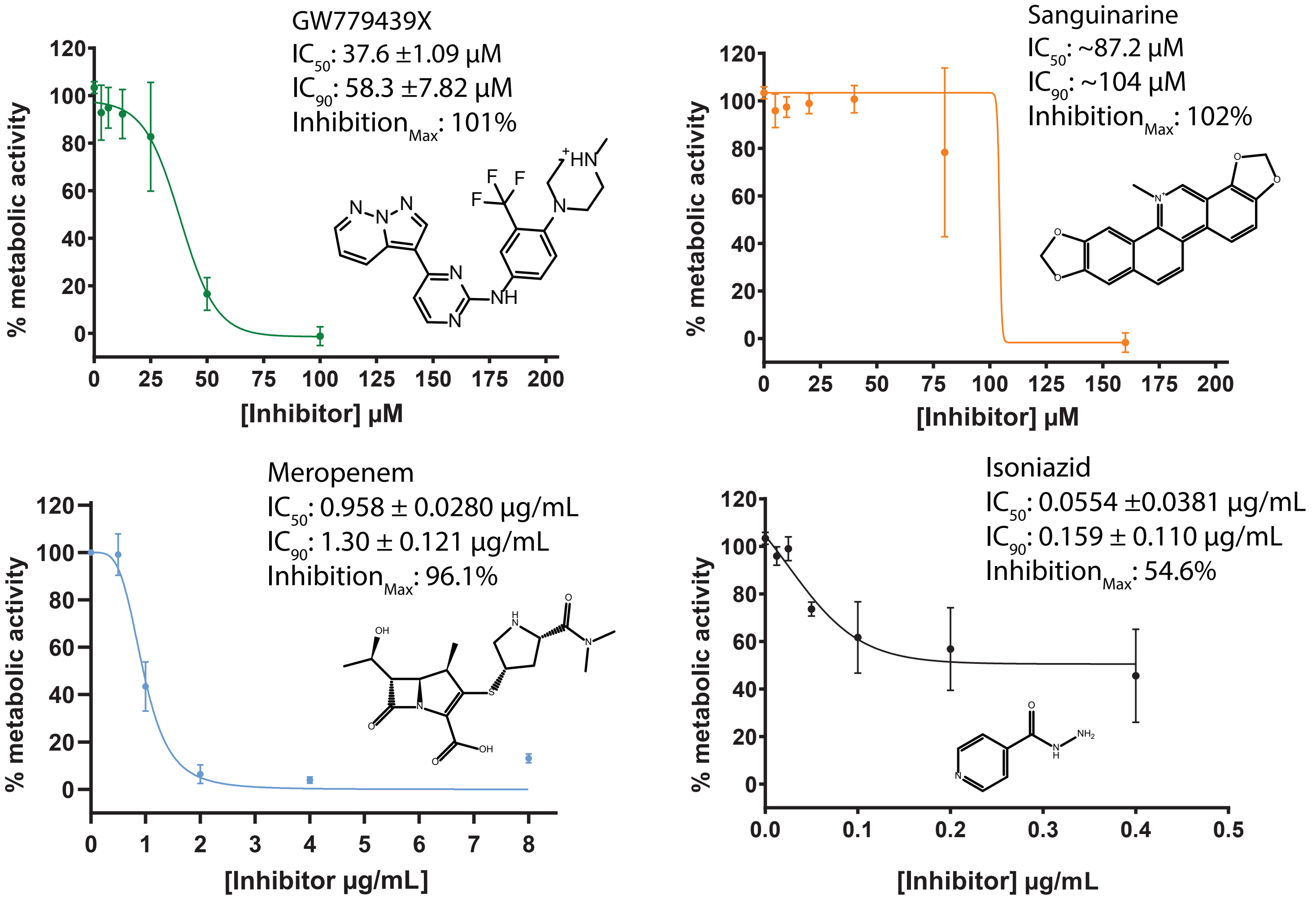

Figure S2: Inhibition curves for small molecules against M. tuberculosis mc²6030. Thirty six of the 126 confirmed PstP inhibitors and relevant control compounds were tested against *M. tuberculosis* mc²6030 in the resazurin assay as described in the methods. **A**: Five PstP inhibitors showed activity against *M. tb* with IC_{90} values between 15 and 200 μM. **B**: Control compounds used for matrix and toxicity experiments all showed inhibition of *M. tb*. Error bars indicate standard deviation and nonlinear regression was used to calculate IC values as described in the methods.

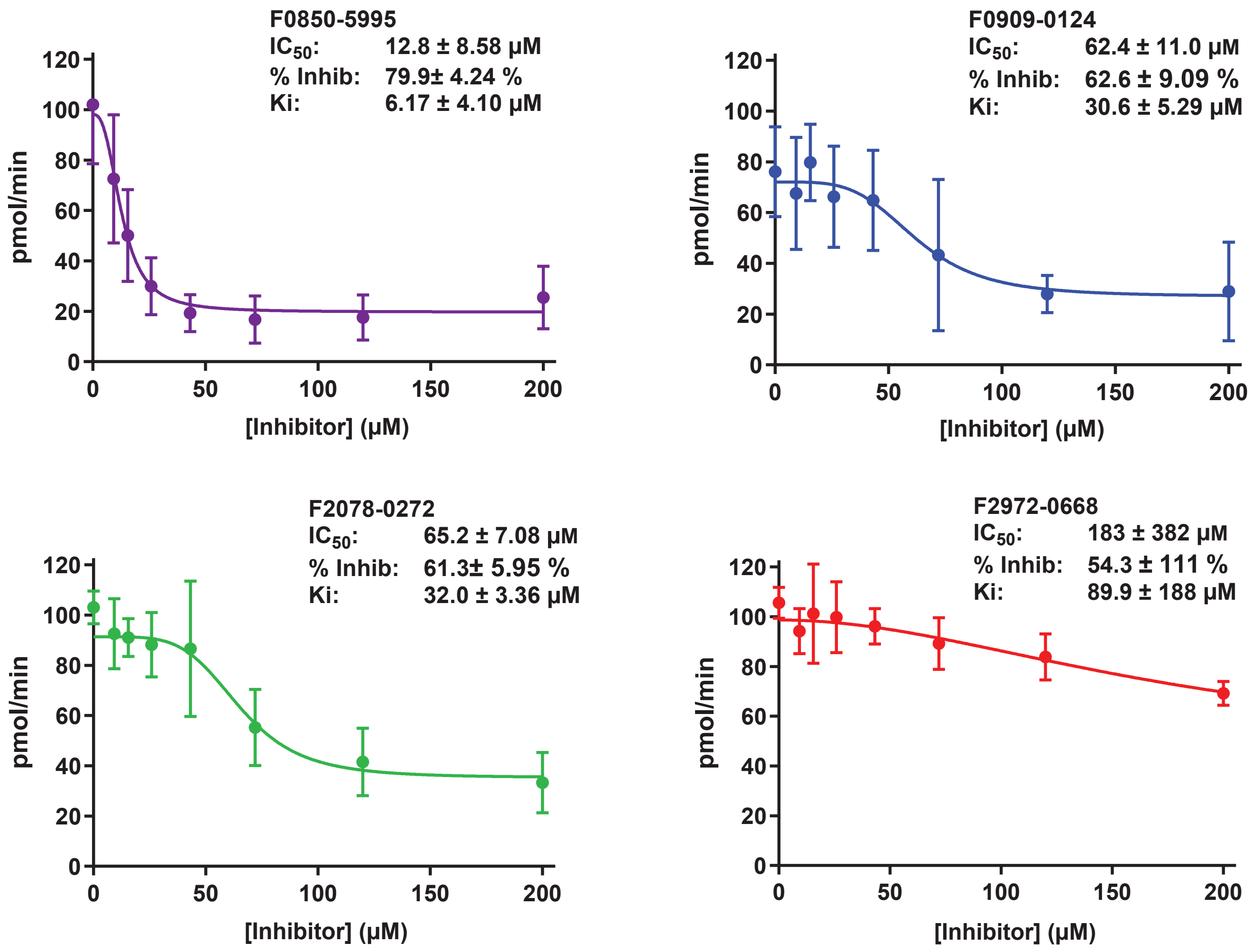

Figure S3: Microbiologically active inhibitors identified in the screen inhibit PstP in a natural substrate biochemical assay. Inhibitors were titrated from 200 to 0 μM and their inhibition of PstP activity on pPknB 1-331 K40A was assessed using the malachite green assay described in the methods. The data were fit with a nonlinear regression variable slope 4 parameter model and plotted. Error bars indicate standard deviation. All four compounds exhibited inhibition, and parameters are indicated; however, compound 2972 had a poor fit since its inhibition was weak therefore parameters have a high degree of error. Data are summarized in **Figure 1**.

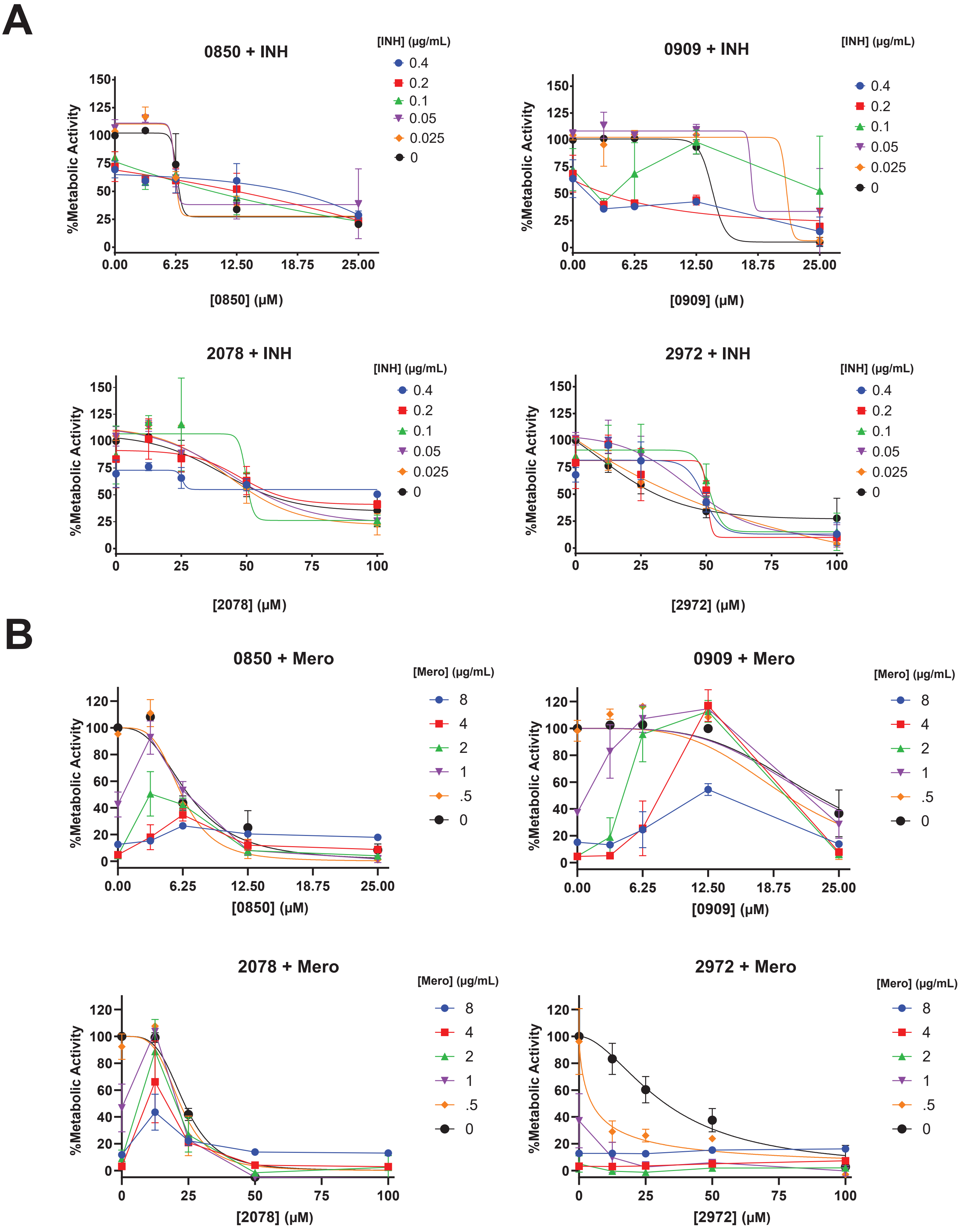

Figure S4: Four microbiologically active PstP inhibitors were titrated in a 2x2 matrix against isoniazid (**A**) or meropenem (**B**). Curves were plotted in Graphpad Prism and IC_{50} and IC_{90} calculated for each curve at each concentration of meropenem or isoniazid. Error bars indicate standard deviation. Data are summarized and compared in **Figure 3**.

A

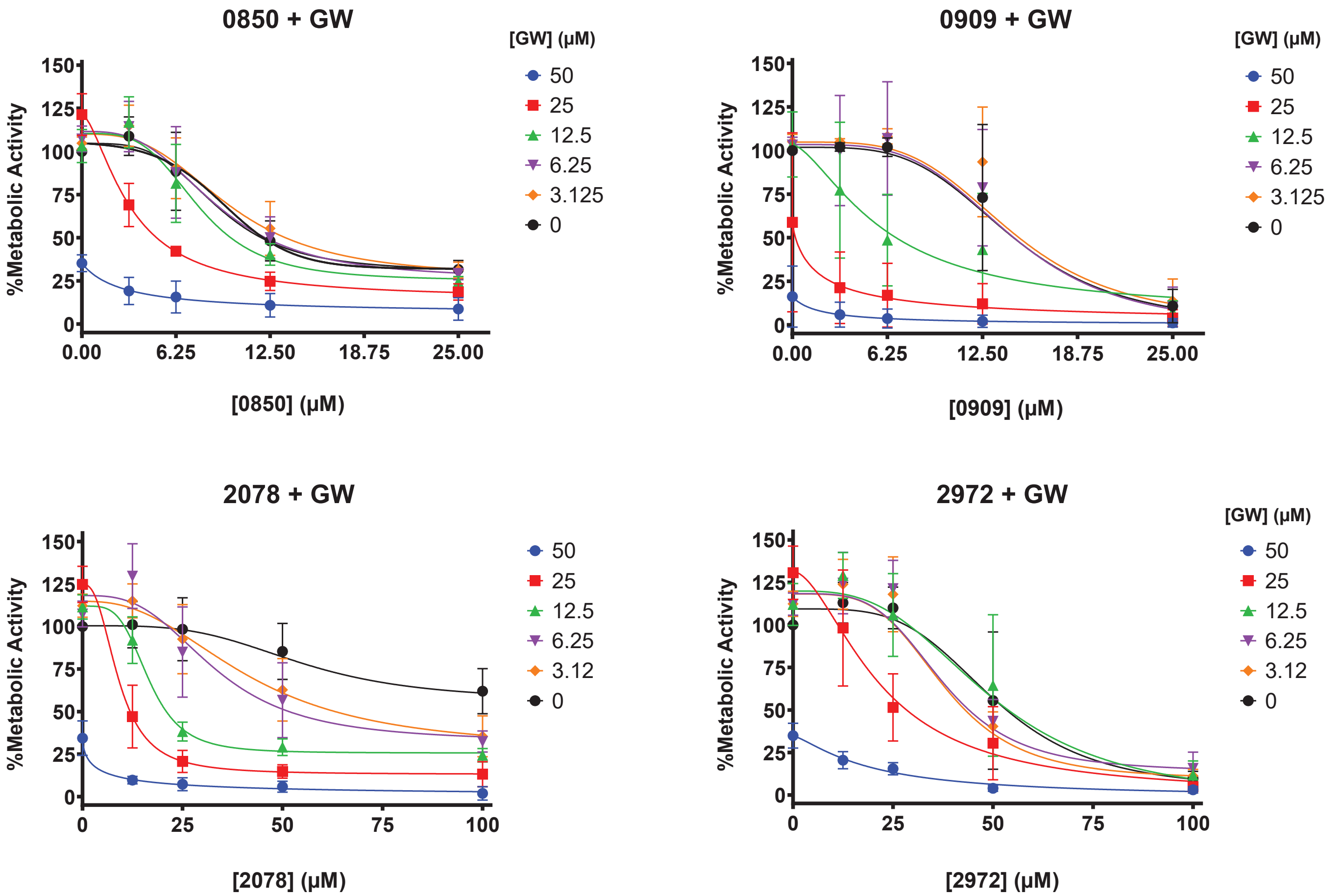
0850 + GW
[GW] (μM)
50
25
12.5
6.25
3.125
0
%Metabolic Activity
150
125
100
75
50
25
0
0.00
6.25
12.50
18.75
25.00
[0850] (μM)
0909 + GW
[GW] (μM)
50
25
12.5
6.25
3.125
0
%Metabolic Activity
[0909] (μM)
2078 + GW
50
25
12.5
6.25
3.12
0
%Metabolic Activity
0
25
50
75
100
[2078] (μM)
2972 + GW
[GW] (μM)
50
25
12.5
6.25
3.12
0
%Metabolic Activity
[2972] (μM)

B

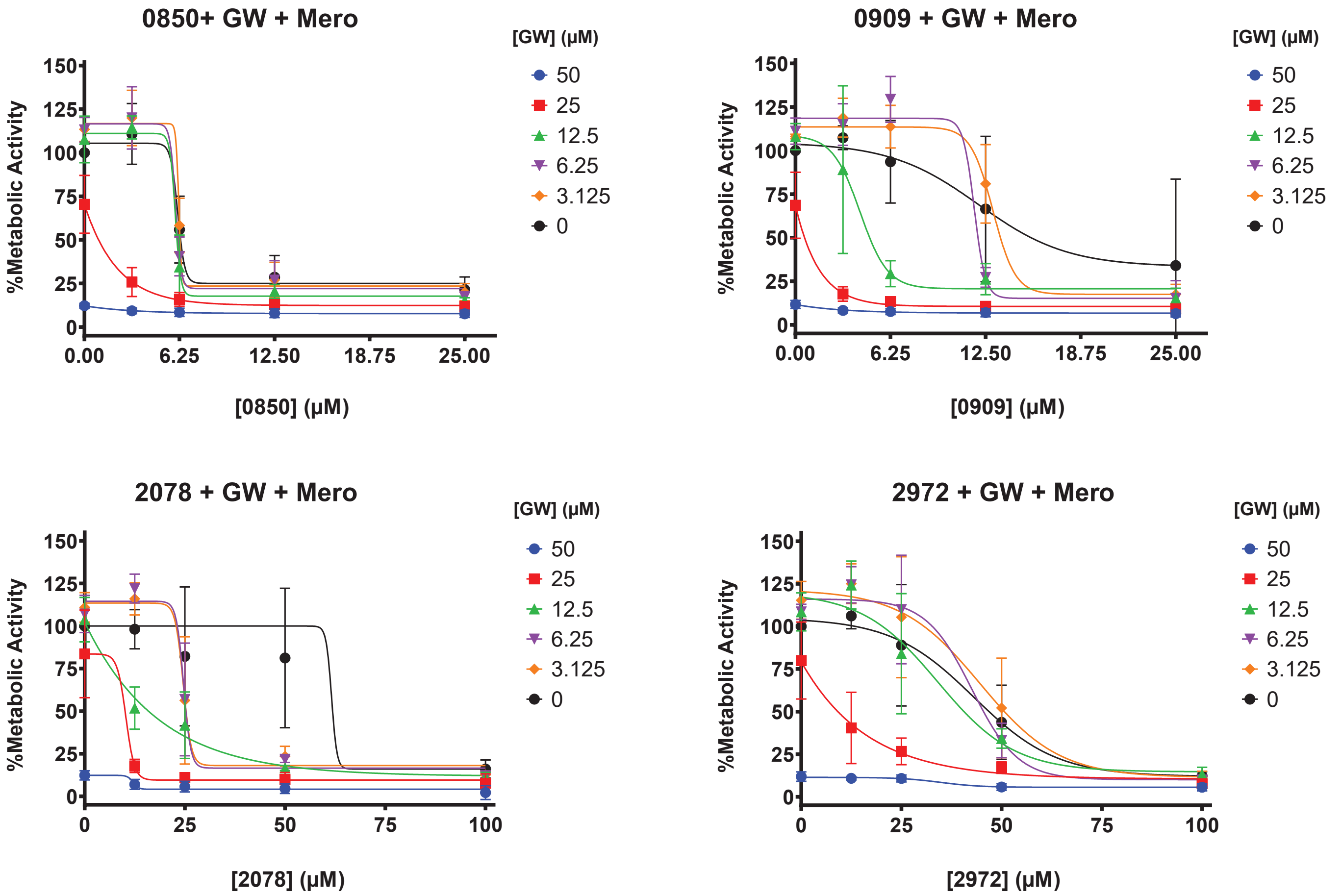
0850+ GW + Mero
[GW] (μM)
50
25
12.5
6.25
3.125
0
%Metabolic Activity
0.00
6.25
12.50
18.75
25.00
[0850] (μM)
0909 + GW + Mero
[GW] (μM)
50
25
12.5
6.25
3.125
0
%Metabolic Activity
[0909] (μM)
2078 + GW + Mero
[GW] (μM)
50
25
12.5
6.25
3.125
0
%Metabolic Activity
0
25
50
75
100
[2078] (μM)
2972 + GW + Mero
[GW] (μM)
50
25
12.5
6.25
3.125
0
%Metabolic Activity
[2972] (μM)

C

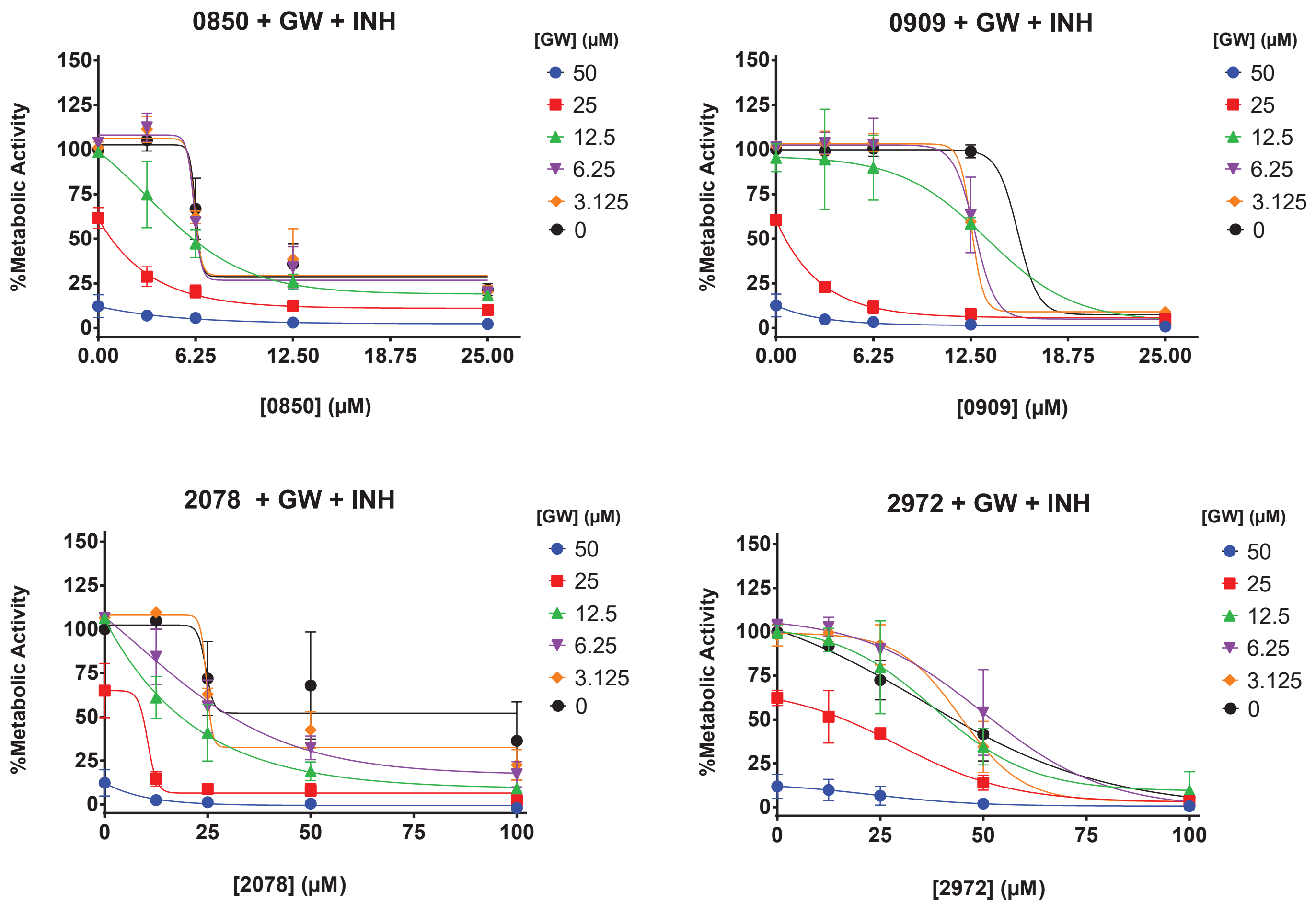

Figure S5: The four microbiologically active PstP inhibitors were titrated in a 2x2 matrix against PknB inhibitor GW779439X (GW) either alone (**A**) or with a sublethal dose (0.5 μg/mL) of meropenem (**B**) or isoniazid (0.05 μg/mL) (**C**). Curves were plotted in Graphpad Prism and IC_{50} and IC_{90} calculated for each curve at each concentration of GW. Error bars indicate standard deviation. Data are summarized and compared in **Figures 1 & 4**.

A

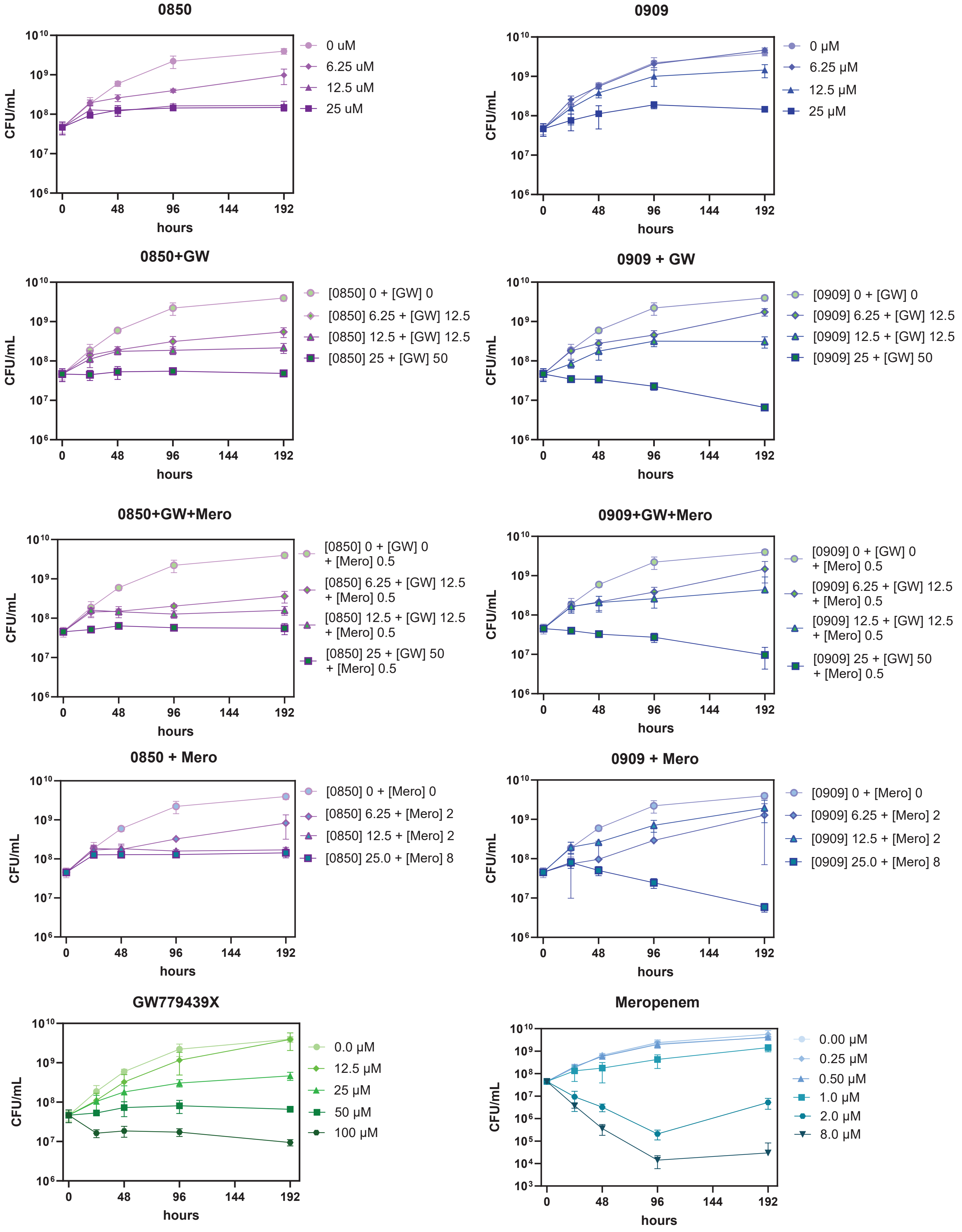
0850
CFU/mL
hours
0 uM
6.25 uM
12.5 uM
25 uM
0909
0 μM
6.25 μM
12.5 μM
25 μM
0850+GW
[0850] 0 + [GW] 0
[0850] 6.25 + [GW] 12.5
[0850] 12.5 + [GW] 12.5
[0850] 25 + [GW] 50
0909 + GW
[0909] 0 + [GW] 0
[0909] 6.25 + [GW] 12.5
[0909] 12.5 + [GW] 12.5
[0909] 25 + [GW] 50
0850+GW+Mero
[0850] 0 + [GW] 0 + [Mero] 0.5
[0850] 6.25 + [GW] 12.5 + [Mero] 0.5
[0850] 12.5 + [GW] 12.5 + [Mero] 0.5
[0850] 25 + [GW] 50 + [Mero] 0.5
0909+GW+Mero
[0909] 0 + [GW] 0 + [Mero] 0.5
[0909] 6.25 + [GW] 12.5 + [Mero] 0.5
[0909] 12.5 + [GW] 12.5 + [Mero] 0.5
[0909] 25 + [GW] 50 + [Mero] 0.5
0850 + Mero
[0850] 0 + [Mero] 0
[0850] 6.25 + [Mero] 2
[0850] 12.5 + [Mero] 2
[0850] 25.0 + [Mero] 8
0909 + Mero
[0909] 0 + [Mero] 0
[0909] 6.25 + [Mero] 2
[0909] 12.5 + [Mero] 2
[0909] 25.0 + [Mero] 8
GW779439X
0.0 μM
12.5 μM
25 μM
50 μM
100 μM
Meropenem
0.00 μM
0.25 μM
0.50 μM
1.0 μM
2.0 μM
8.0 μM

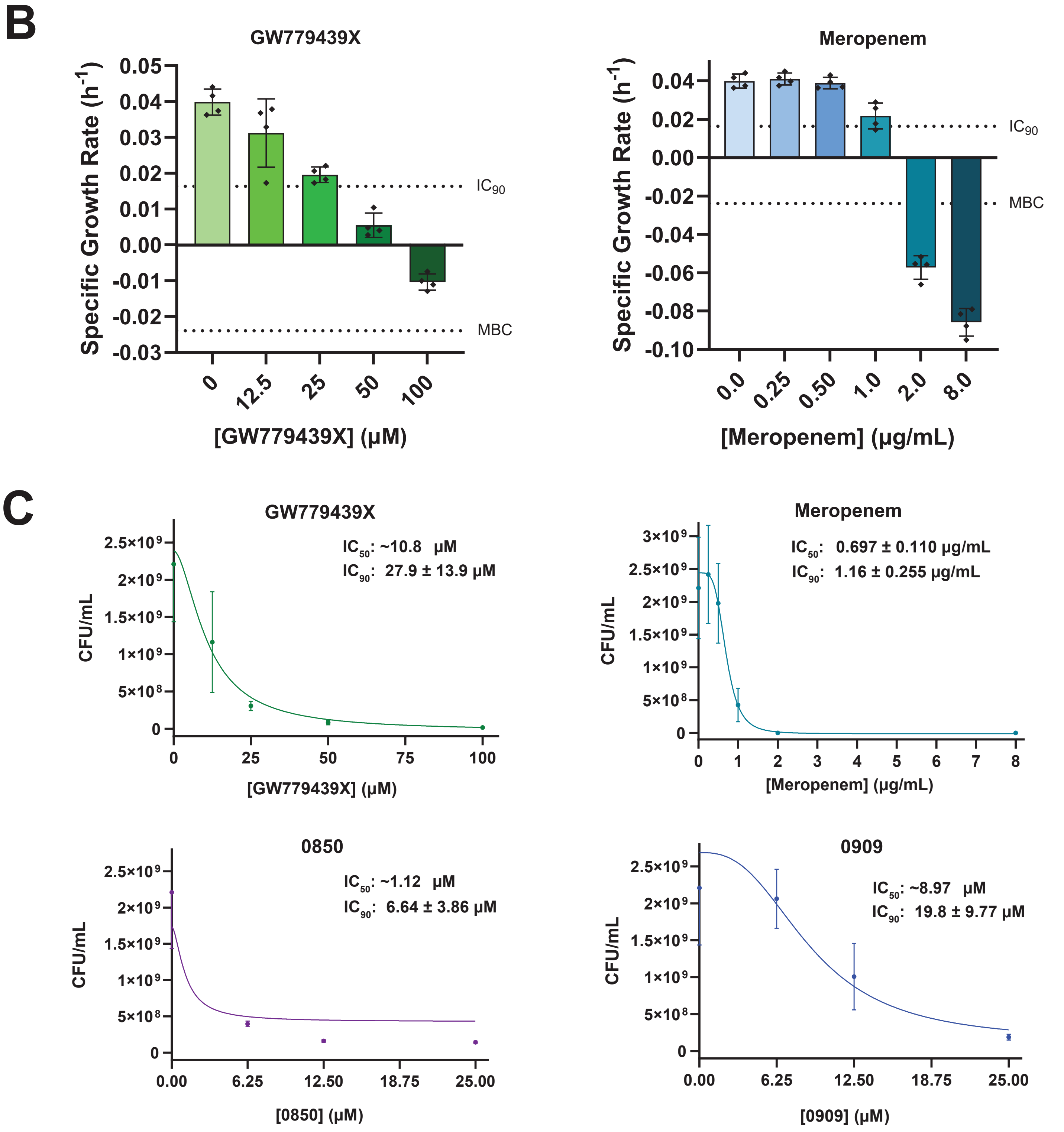

Figure S6: GW, meropenem, and the two most promising PstP inhibitors, 0850 and 0909, were tested against pathogenic *M. tuberculosis* (Erdman). **A**: The PstP inhibitors were tested in the presence and absence of select concentrations of GW, GW with 0.5 μg/mL meropenem, or meropenem alone, and growth and inhibition was measured by OD_{600} and CFU at 0, 24, 48, 96, and 192 hours. **B**: Specific growth rate was calculated for GW779439X and meropenem as described in the methods and plotted to show a dose response. IC_{90} and MBC breakpoints are indicated by dotted lines. **C**: CFU/mL at 96 hours was plotted against inhibitor concentration. IC_{50} and IC_{90} were determined as described in the methods. Error bars indicate standard deviation. Data are summarized and compared in **Figures 1 & 5**.

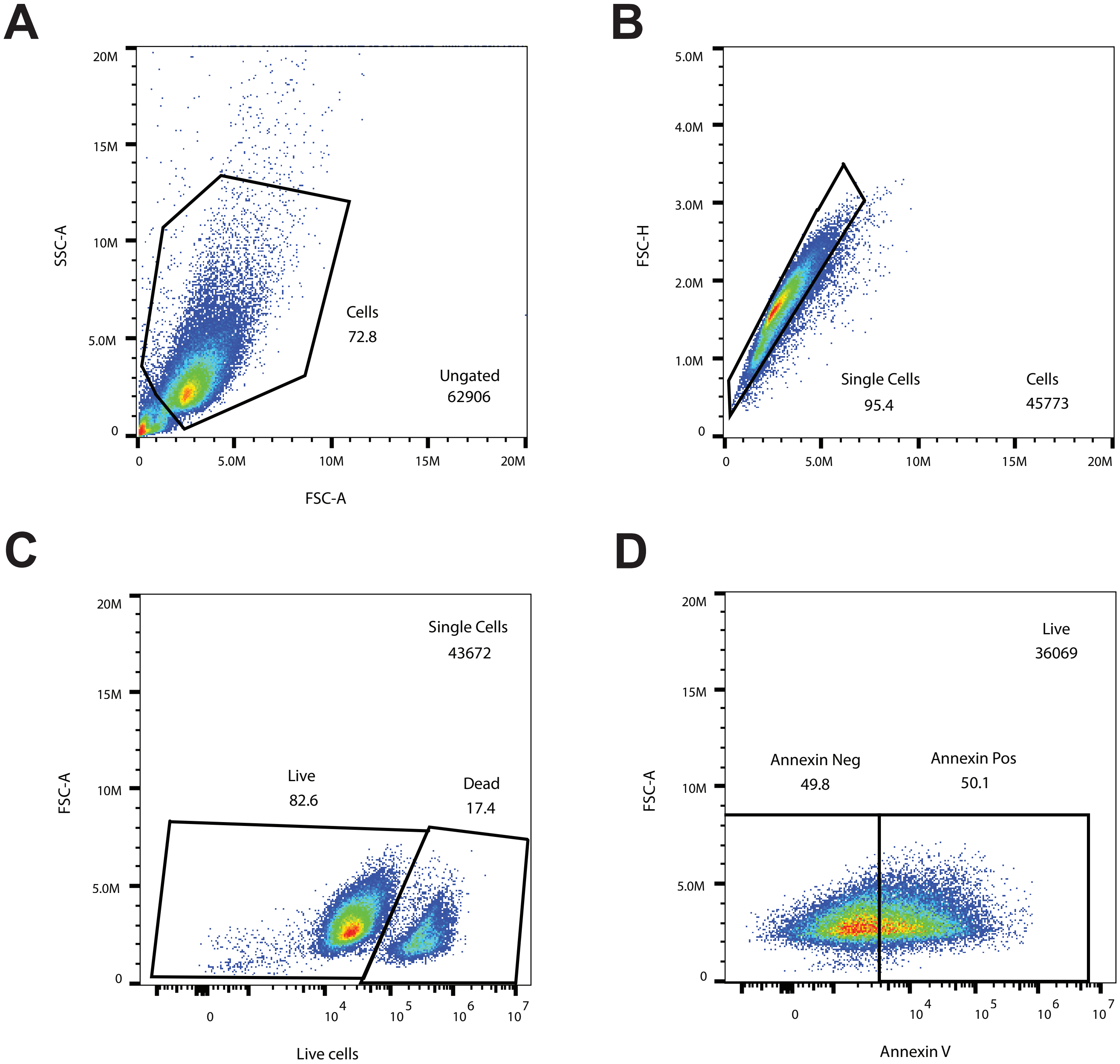

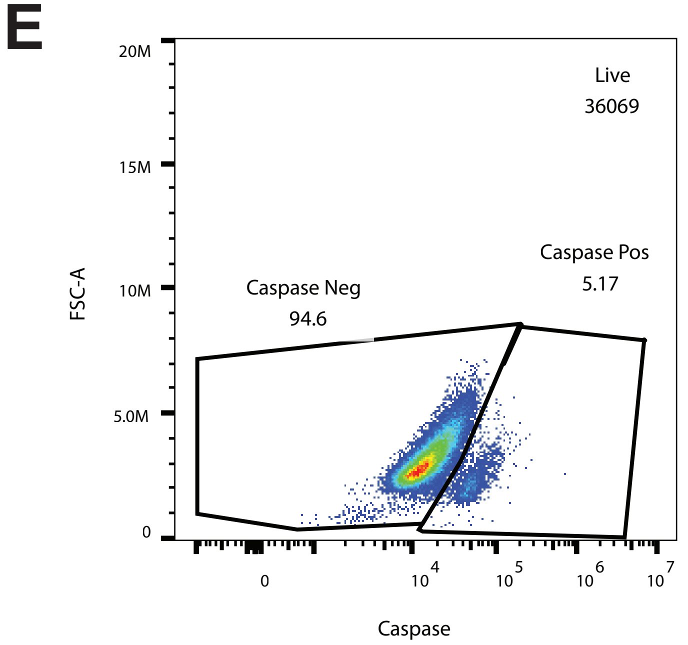

Figure S7: Representative plots for panel A (cytotoxicity) flow cytometry gating strategies. Cells were gated **A**: based on idealized forward and side scatter size, then **B**: to separate aggregates from single cells. **C**: A dye for viability allowed live-dead gating, and **D**: annexin and **E**: caspase dying kits were used to gate cells to identify markers for apoptosis.

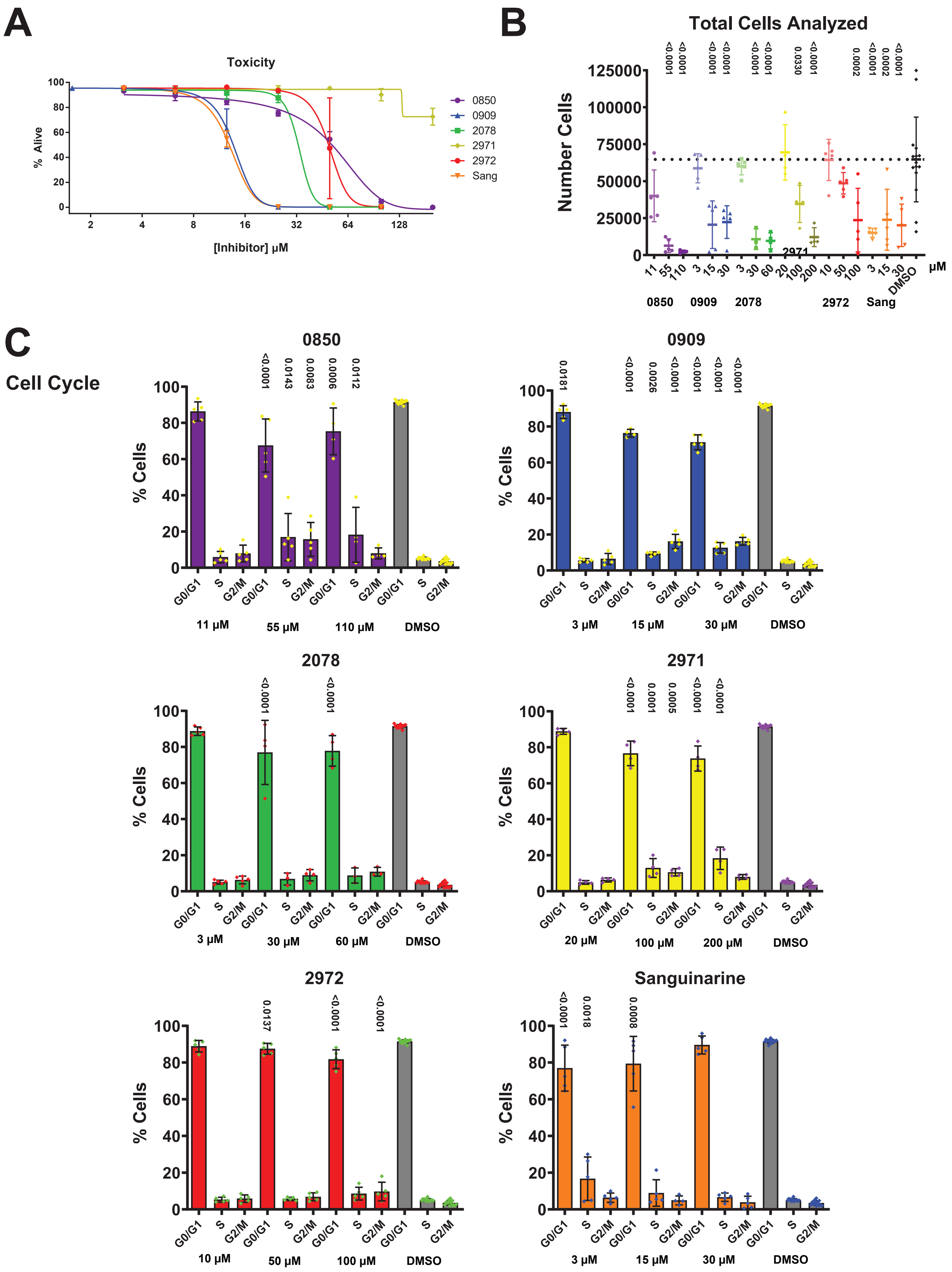
A
Toxicity
% Alive
[Inhibitor] µM
0850
0909
2078
2971
2972
Sang
B
Total Cells Analyzed
Number Cells
µM
0850
0909
2078
2971
2972
Sang
DMSO
C
Cell Cycle
0850
0909
2078
2971
2972
Sanguinarine
% Cells
G0/G1
S
G2/M
11 µM
55 µM
110 µM
DMSO
3 µM
15 µM
30 µM
60 µM
20 µM
100 µM
200 µM
10 µM
50 µM

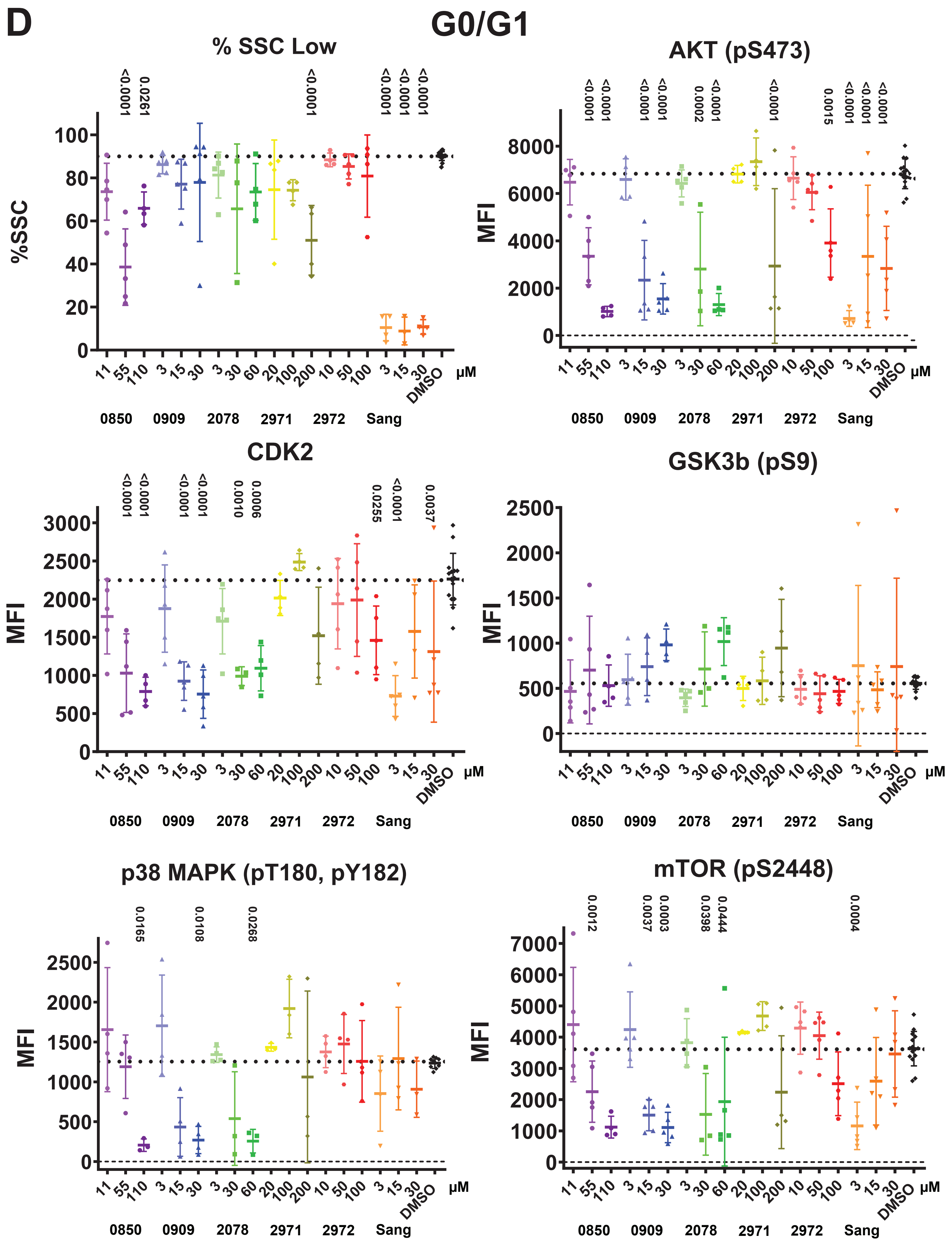
D
G0/G1
% SSC Low
%SSC
AKT (pS473)
CDK2
GSK3b (pS9)
p38 MAPK (pT180, pY182)
mTOR (pS2448)
MFI
0850
0909
2078
2971
2972
Sang
DMSO
µM
<0.001
0.0261
0.0002
0.0015
0.0010
0.0006
0.0255
0.0037
0.0165
0.0108
0.0268
0.0012
0.0037
0.0003
0.0398
0.0444
0.0004

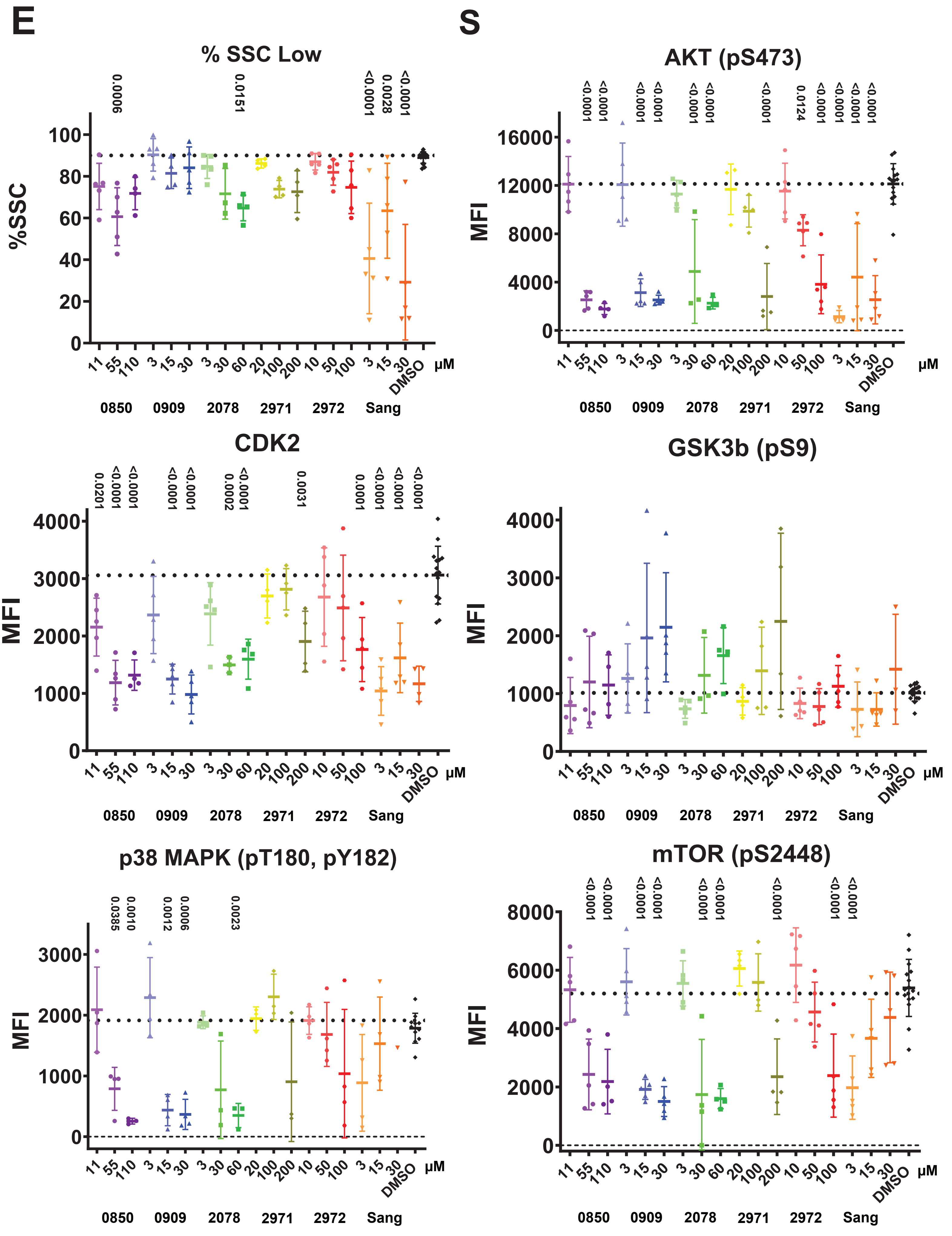
E
S
% SSC Low
%SSC
0.0006
0.0151
<0.0001
0.0028
<0.0001
AKT (pS473)
MFI
<0.0001
0.0124
CDK2
0.0201
0.0002
0.0031
0.0001
GSK3b (pS9)
p38 MAPK (pT180, pY182)
0.0385
0.0010
0.0012
0.0006
0.0023
mTOR (pS2448)
11 55 110 3 15 30 3 30 60 20 100 200 10 50 100 3 15 30 DMSO μM
0850
0909
2078
2971
2972
Sang

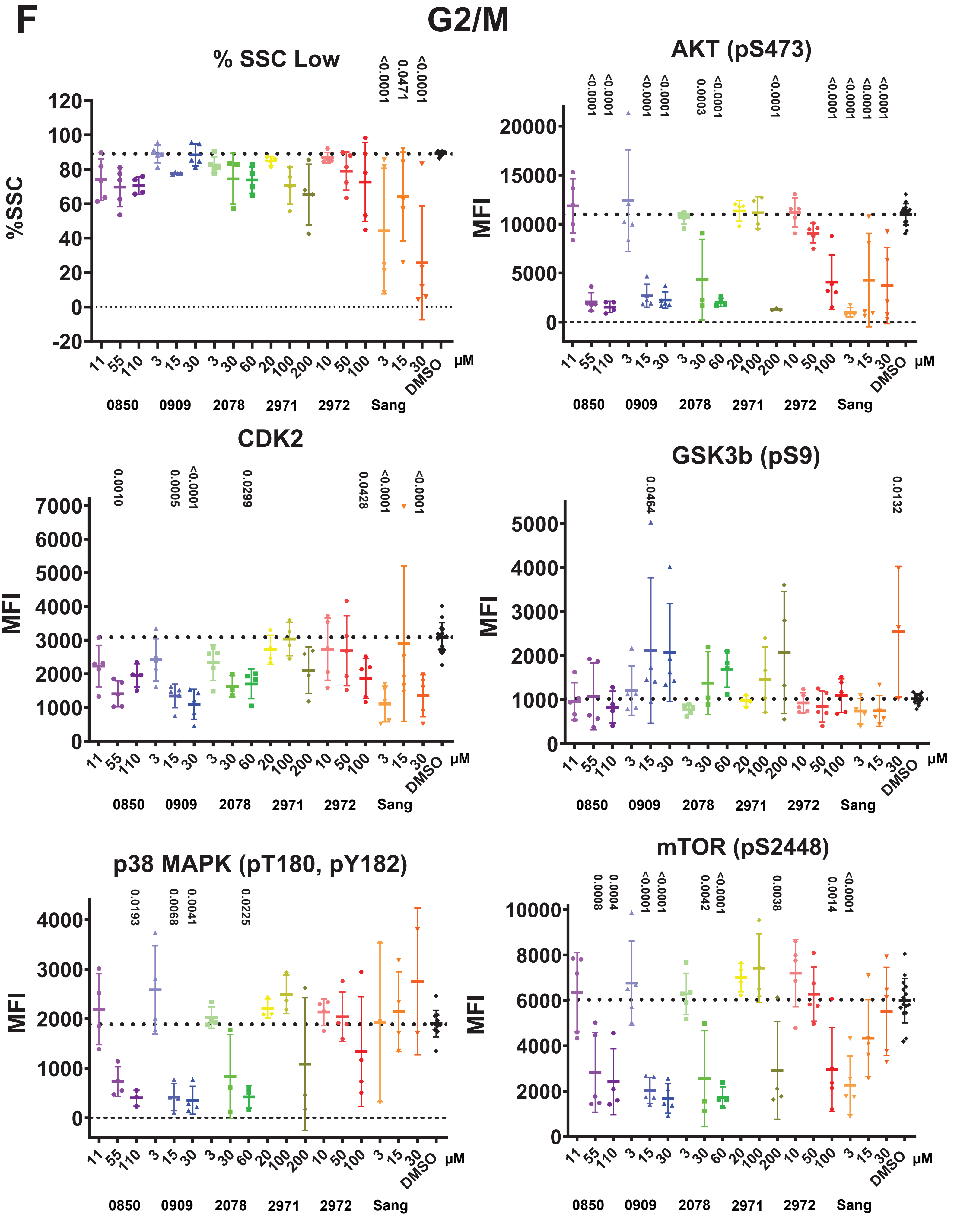
F
G2/M
% SSC Low
%SSC
<0.0001
0.0471
<0.0001
AKT (pS473)
MFI
<0.0001
<0.0001
<0.0001
<0.0001
0.0003
<0.0001
<0.0001
<0.0001
<0.0001
<0.0001
<0.0001
CDK2
MFI
0.0010
0.0005
<0.0001
0.0299
0.0428
<0.0001
<0.0001
GSK3b (pS9)
MFI
0.0464
0.0132
p38 MAPK (pT180, pY182)
MFI
0.0193
0.0068
0.0041
0.0225
mTOR (pS2448)
MFI
0.0008
0.0004
<0.0001
<0.0001
0.0042
<0.0001
0.0038
0.0014
<0.0001
11 55 110 3 15 30 3 30 60 20 100 200 10 50 100 3 15 30 DMSO μM
0850 0909 2078 2971 2972 Sang

Figure S8: PstP inhibitors induce cell cycle and key phospho-protein changes at or above their CC_{50} in THP-1 human monocytes. **A**: Cytotoxicity was assessed in panel A as described in the methods and graphed for a dose response. Compound data for all subsequent panels is shown from flow cytometry panel B (cell cycle regulation). Each compound was tested at three concentrations (1/5 CC50, CC50, and 2x CC50) to correspond approximately to the upper and lower inflection points and center of the cytotoxicity curve. For all subsequent panels, significance is indicated (vertically for space) with P-values for each comparison and absence of a value above is non-significant. Data for compounds 0850, 0909, and sanguinarine on cell cycles and markers in G0 phase from **Figure 6 C & D** are repeated here for comparison to other compounds. **B**: Total cells analyzed for each compound dose in panel B. **C**: Changes in cell cycle stages induced by each compound were analyzed and compared to vehicle (DMSO) control. Significance from DMSO (dotted line) for each stage was determined by one-way ANOVA with Sidak's multiple comparisons test for paired columns. Due to cytotoxicity at higher doses, the total number of live cells analyzed were compared to DMSO similarly by using one way ANOVA and Dunnet's test for multiple comparisons to control. **D**: Analysis of phospho-proteins which regulate the cell cycle show differences in G0/G1 phase at or above CC_{50}. Changes in cell shape by granularity (% side scatter low) were compared as well as differences in mean fluorescence intensity (MFI) of five markers: Akt pS437, Cdk2 (total), Gsk3β pS9, p38 MAPK pT180 & pY182, and mTOR pS2448. Significance from DMSO was determined by one-way ANOVA and Dunnet's test for multiple comparisons. A dashed line was used to indicate baseline (0) where error bars extended below baseline. Data from the same analysis is shown for cells in S phase (**E**) and G2/M phase (**F**). Individual data points are shown along with the mean and standard deviation for all graphs.

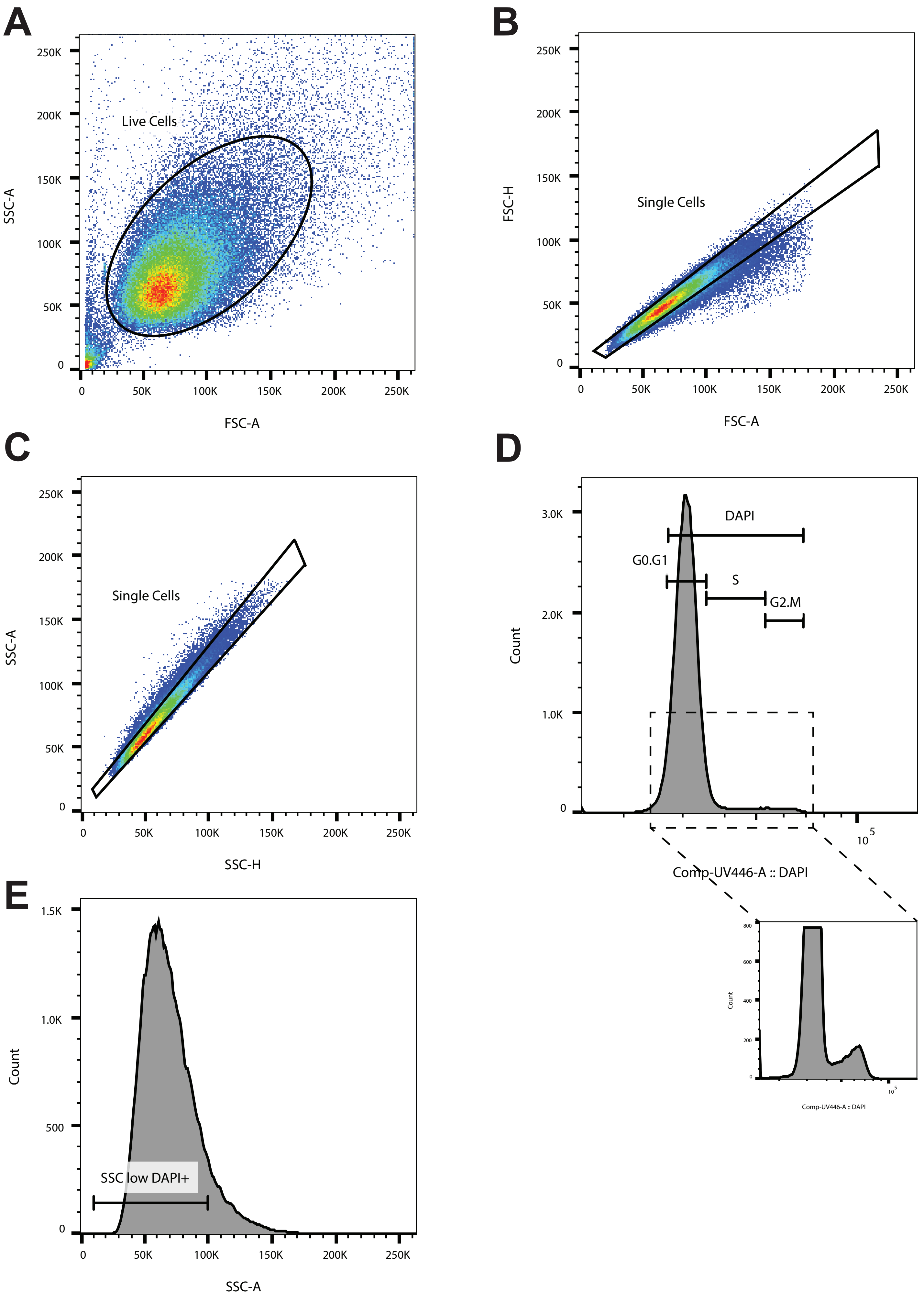
A
Live Cells
SSC-A
FSC-A
250K
200K
150K
100K
50K
0
B
Single Cells
FSC-H
FSC-A
C
Single Cells
SSC-A
SSC-H
D
DAPI
G0.G1
S
G2.M
Count
3.0K
2.0K
1.0K
10 5
Comp-UV446-A :: DAPI
800
600
400
200
E
SSC low DAPI+
Count
1.5K
1.0K
500
SSC-A

F

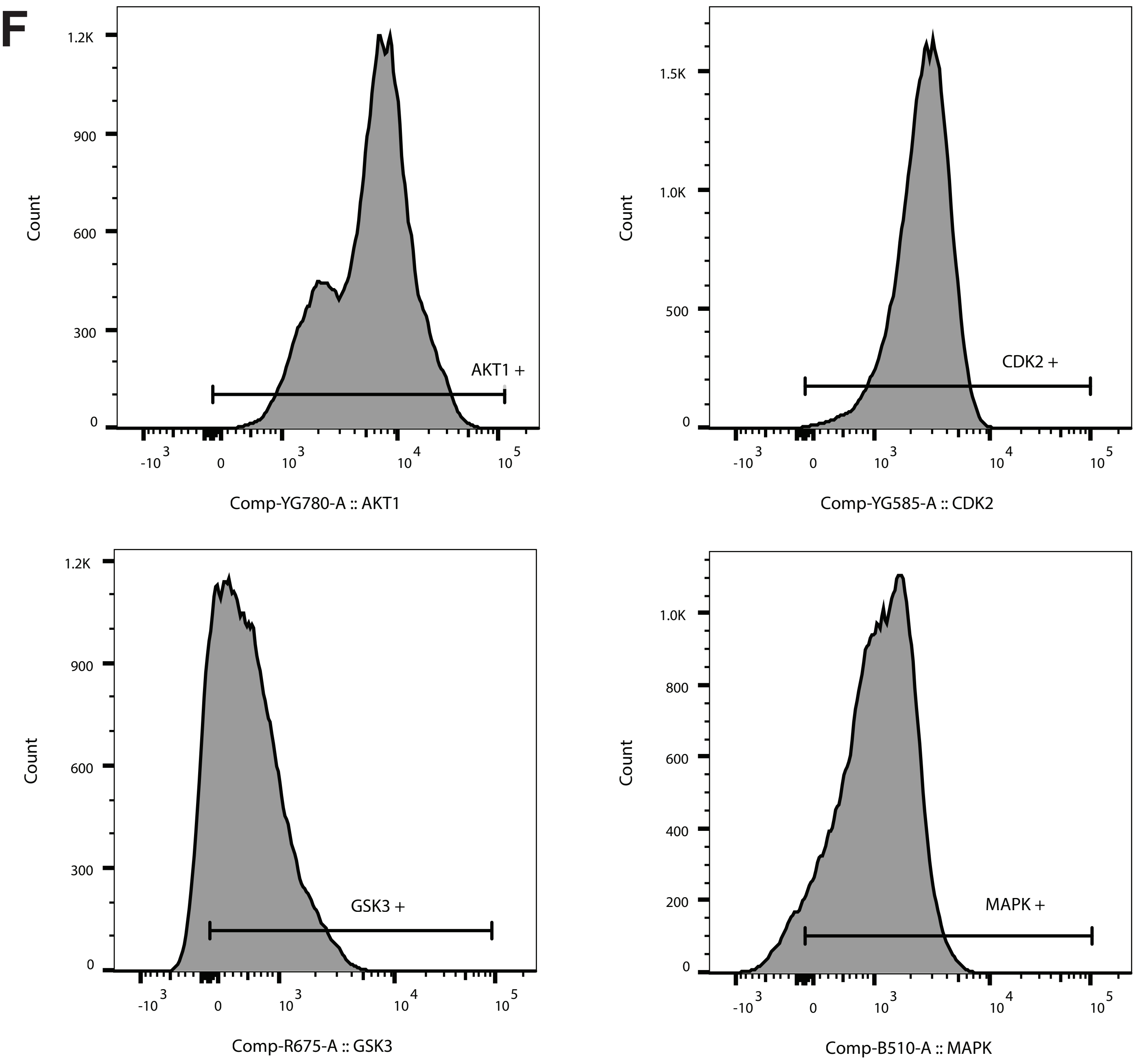

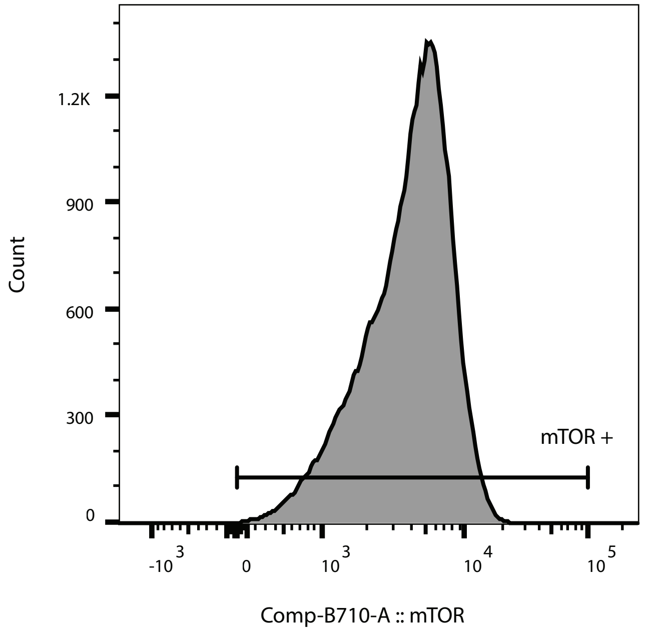

Figure S9: Representative plots for panel B (cell cycle regulation) flow cytometry gating strategies. Cells were gated **A**: based on idealized forward and side scatter size, then **B**: to separate aggregates from single cells on forward scatter and then **C**: side scatter. DAPI stain on the cells **D**: was assessed and gated based on cell cycle stage. **E**: Cells used for analysis were limited to DAPI positive with low (below 100,000) side scatter. **F**: Representative gates for each antibody (Akt pS437, Cdk2 (total), Gsk3β pS9, p38 MAPK pT180 & pY182, and mTOR pS2448) are shown and were drawn based on positive signal using fluorescence minus one (FMO) comparisons.
